# An Interacting Quantum Atoms (IQA) and Relative Energy Gradient (REG) Analysis of the Anomeric Effect

**DOI:** 10.3390/molecules27155003

**Published:** 2022-08-06

**Authors:** Danish Khan, Leonardo J. Duarte, Paul L. A. Popelier

**Affiliations:** 1 Faculty of Physics, University of Vienna, Kolingasse 14-16, 1090 Vienna, Austria; 2 Institute of Chemistry, State University of Campinas, Campinas 13083-970, SP, Brazil; 3 Department of Chemistry, The University of Manchester, Manchester M13 9PL, UK

**Keywords:** Relative Energy Gradient (REG), Interacting Quantum Atoms (IQA), Quantum Theory of Atoms in Molecules (QTAIM), anomeric effect, dimethoxymethane, 2-fluorotetrahydropyran

## Abstract

The explanation of the anomeric effect in terms of underlying quantum properties is still controversial almost 70 years after its introduction. Here, we use a method called Relative Energy Gradient (REG), which is able to compute chemical insight with a view to explaining the anomeric effect. REG operates on atomic energy contributions generated by the quantum topological energy decomposition Interacting Quantum Atoms (IQA). Based on the case studies of dimethoxymethane and 2-fluorotetrahydropyran, we show that the anomeric effect is electrostatic in nature rather than governed by hyperconjugation.

## 1. Introduction

The anomeric effect is a well-known and important stereoelectronic effect altering the conformational preferences of various heterocyclic systems, which was first observed [1] in pyranose rings by Edward in 1955. The term was later coined [2] by Lemieux and Chu in 1958 while also studying carbohydrate chemistry. The effect was originally defined as the anomalous preference of an electronegative substituent at an anomeric carbon in a glucopyranose ring to adopt an axial position rather than an equatorial position. The anomaly arises due to the tendency of substituents to adopt the equatorial position in substituted cyclohexanes, as expected from steric considerations. The anomeric effect has since been observed in saturated heterocyclic compounds with a heterosubstituent group adjacent to the heteroatom and in acyclic compounds with electronegative heteroatoms [3,4,5,6]. However, the cause behind this effect remains a subject of debate, with several disparate explanations, and hitherto without consensus [7,8,9,10,11,12,13,14,15].

Currently, the most widely accepted explanation relies on a stabilizing hyperconjugation interaction between the lone pair of the endocyclic oxygen (O) atom and the vacant antibonding σ* orbital of the adjacent carbon (C1) atom, to which the substituent heteroatom (X) is attached [7,13]. The axial conformation is then favoured by the lower energy following from the antiperiplanar alignment between the donating lone pair and the exocyclic C-X σ bond. This interaction is supported by the observation that the C-O bond length decreases while the C-X bond length increases in compounds that display the anomeric effect [15], with further support from early theoretical studies based upon NBO analysis [13]. However, this mechanism has been criticized by several recent theoretical studies [8,9,10]. Mo used [8] the Block-Localized Wavefunction (BLW) approach to ‘quench’ the hyperconjugation effects from dimethoxymethane and substituted tetrahydropyrans. That work showed that the anomeric effect persisted (and in fact increased in magnitude) even after all intramolecular electron delocalization was quenched. Mo concluded that “BLW computations do not support the currently popular hyperconjugation explanation for the anomeric effect”, and his energy analysis indicates that steric and electrostatic effects instead dominate the conformational preference. In line with this finding, Vila and Mosquera used [9] the original QTAIM virial-based energy partitioning, as well as its atomic population and delocalization index, to study dimethoxymethane and methanediol. They concluded that “values of delocalization indexes and atomic electron populations demonstrate that the OCO anomeric effect cannot be rationalized as due to electron delocalizations. In contrast, the examination of electron atomic populations and geometries allows explaining it on the basis of repulsive and attractive interactions”, with the note that QTAIM assigns a steric origin to the effect.

The dipole moment alignment hypothesis first put forward by Edward was subsequently developed into a more comprehensive electrostatic model by Lemieux and Chu, which involved a [1,2,3,4] Coulombic interaction between the axial substituent (X) and the other (substituent-free) carbon (C2) atom adjacent to O. Recently, this mechanism has been further revised [11,12] by the inclusion of a stabilizing nonclassical CH…X hydrogen bond interaction between the axial substituent and the syn-axial hydrogen atom attached to C2. This interaction was studied [11] in detail recently by Wiberg et al. through a combined experimental and computational study, leading them to conclude that “the anomeric effect appears to arise mainly from two separate CH heteroatom non-bonded Coulombic attractions; in a sense, non-classical hydrogen bonds”. An interesting and relevant point made in this study through MP2/aug-cc-pVTZ calculations, which has also been pointed out by Vila and Mosquera and by Wang et al., is the significant charge shift from the hydrogen atoms to the C2 carbon atoms. This shift leads to enhanced Coulombic interaction between these equatorial hydrogen atoms and the ring heteroatom.

However, in contrast to these studies, Silva et al. provided [14] evidence that the anomeric effect is dominated by differences in exchange energy and that it has no electrostatic origin. Their conclusion was based on a Hartree–Fock energy decomposition analysis of substituted cyclohexane and tetrahydropyran-2-yl molecules at the HF/6-31G(d,p) level. They also showed that the MP2/CCSD correlation correction was small. Meanwhile, Liu et al. found [16] a middle ground in their conclusion: that a combination of classical Coulomb interaction and exchange-correlation interaction plays the greatest role in the anomeric effect. They reached this conclusion through a statistical analysis of 80 axial–equatorial conformation pairs of α-D-glucopyranose from partitioned energy components obtained at the B3LYP/6-311+G(d) level [40,41].

The above account makes clear how convoluted this longstanding debate is. The contradictory and thus inconclusive nature of these results calls for an exhaustive analysis using a more modern energy partitioning method [17] called interacting quantum atoms (IQA). This method was inspired by earlier work [18] carried out in 2001 introducing a quantum topological energy partitioning that is also valid at non-stationary points (i.e., non-equilibrium geometries) on the potential energy surface (PES). This was an important extension to the previous virial-based energy partitioning the standard in the Quantum Theory of Atoms in Molecules (QTAIM) [19,20,21] because the latter is confined to stationary points. As an orbital-free and parameter-free energy partitioning scheme, IQA allows for each type of energy contribution (kinetic, exchange-correlation, and electrostatics) of a system to be calculated in a well-defined manner. Admittedly, at a practical level, QTAIM introduces a numerical error caused by the quadrature executing the integration over the volumes of the topological atoms. However, this error is typically of the order of 1–2 kJ∙mol^−1^ such that the sum of all energy contributions recovers to a sufficient degree the total energy of the wavefunction. The well-separated nature of the various IQA energy contributions is an asset compared to the various partitioning schemes used in non-topological energy partitioning studies. This advantage makes it possible to study the electrostatic, exchange, electron correlation [22], and even kinetic energy terms in a transparent and convincing way. To deal with the many energy contributions arising in the comprehensive analysis of the current study, the recently developed Relative Energy Gradient (REG) method [23] will also be used in our analysis.

As the model for the anomeric effect in this article, we studied two compounds: dimethoxymethane (DMM) (which is acyclic) and 2-fluorotetrahydropyran (FTHP) (which is cyclic). DMM is the simplest molecule displaying the anomeric effect with its gauche-gauche conformer being about 26 kJ∙mol^−1^ more stable than its trans-trans conformer [24], which is attributable to the anomeric effect; this difference is about twice as large as that in sugars [8]. This makes DMM the perfect prototype for the anomeric effect, which is why it has been the subject of several such theoretical studies [8,9,24,25].

Due to the anomeric effect, the axial conformation of 2-fluorotetrahydropyran is about 12–14 kJ∙mol^−1^ more stable than the equatorial conformation [8,26], which is among the largest differences in substituted tetrahydropyrans and substituted cyclohexanes [8,26]. This fact makes FTHP an attractive molecule to study. Moreover, Tschumper et al. conducted [26] a study comparing the impact on this anomeric energy difference due to the level of theory used (i.e., method and basis set). They reported that, while the B3LYP method is generally unreliable compared to MP2 and CCSD(T) (even with very diffuse basis sets), the energy difference between B3LYP and MP2/CCSD(T) is the least in the case of FTHP among all the other substituted tetrahydropyrans. They attributed this to a fairly consistent cancellation of errors in this case. This fact justifies proceeding with B3LYP, which was made compatible [27] with IQA for the first time in 2016, rather than with MP2 or CCSD(T), which require vastly higher computational expense. In summary, these two facts make FTHP another viable prototype for our study, and hence, it too was chosen alongside DMM. The comparison between these two molecules will also help us to verify if the same mechanism can be generalized to both cyclic and acyclic molecules.

## 2. Background

### 2.1. Interacting Quantum Atoms (IQA)

This method has been explained many times before, so we only give a brief overview here in order to introduce the nomenclature used in the Results and Discussions section and the theory behind the interpretation of those results.

Since topological atoms are space-filling (i.e., no overlap and no gaps), their properties are additive. So, the total energy of the system can simply be recovered, with a small integration error, by adding the various atomic energy components. In principle, IQA considers four energy types as defined in Equation (1),

(1) $$E_{\mathrm{total}}=\overset{\mathrm{atoms}}{\underset{A}{\sum }}E_{\mathrm{intra}}\left(A\right)+\frac{1}{2}\overset{\mathrm{atoms}}{\underset{B\neq A}{\sum }}{\;V}_{\mathrm{cl}}\left(A,B\right)+V_{x}\left(A,B\right)+V_{c}\left(A,B\right)$$

where $E_{\mathrm{intra}}\;$ contains all the energetic contributions within an atomic basin and $V_{\mathrm{cl}}\left(A,B\right)$ represents the classical (cl) electrostatic energy between the atoms A and B (henceforth referred to as “electrostatics”). The term $V_{x}\left(A,B\right)$ is the exchange energy between atoms A and B, while $V_{c}\left(A,B\right)$ is the correlation energy. Density functional theory makes the exchange energy join the correlation energy in a single term $V_{\mathrm{xc}}\left(A,B\right)$, which is the energy we proceed with in the current study. Hence, only three IQA components of Equation (1) will appear from hereon.

The term $V_{\mathrm{cl}}\left(A,B\right)$ is introduced because the Coulomb term is typically not analysed as a pure electron–electron energy term, $V_{\mathrm{ee},\mathrm{coul}}\left(A,B\right),$ but is combined with potential energies involving the nuclear charges of atoms A and B, or

(2) $$V_{\mathrm{cl}}\left(A,B\right)=V_{\mathrm{ee},\mathrm{coul}}\left(A,B\right)+V_{\mathrm{en}}\left(A,B\right)+V_{\mathrm{ne}}\left(A,B\right)+V_{\mathrm{nn}}\left(A,B\right)$$

where $V_{\mathrm{ne}}\left(A,B\right)$ is the energy associated with the nucleus of atom A interacting with the electron density of atom B, and $V_{\mathrm{nn}}\left(A,B\right)$ is the nucleus-nucleus repulsion.

The $E_{\mathrm{intra}}$ term consists of all the intra-atomic potential energy as well as kinetic energy,

(3) $$E_{\mathrm{intra}}\left(A\right)=T\left(A\right)+V_{\mathrm{cl}}\left(A,A\right)+V_{\mathrm{xc}}\left(A,A\right)$$

where $T\left(A\right)$ is the (intra-atomic) kinetic energy, and $V_{\mathrm{cl}}\left(A,A\right)$, the intra-atomic counterpart of $V_{\mathrm{cl}}\left(A,B\right)$, is given by

(4) $$V_{\mathrm{cl}}\left(A,A\right)=V_{\mathrm{ee},\mathrm{coul}}\left(A,A\right)+{\;V}_{\mathrm{en}}\left(A,A\right)$$

Changes in $E_{\mathrm{intra}}$ indicate changes in the internal electron density of the atomic basin, and it has been shown [28,29] that there is a quantitative link between steric repulsion and $E_{\mathrm{intra}}$ via the latter’s successful fitting to the well-known exponential Buckingham potential.

### 2.2. Relative Energy Gradient (REG) Method

The REG method [23] has also been explained several times before, with a growing number of studies such as on the fluorine gauche effect [30], the reaction mechanism and catalytic effects in the peptide hydrolysis [31] in HIV-1 protease, the rotation barrier [32] in biphenyl, or the halogen bond with explicit analysis of electron correlation [33]. REG is associated with a dynamic change to the system under study. The method thus needs a sequence of “snapshots” of the system in order to display to what extent a given IQA energy term’s profile follows the total energy profile. This sequence is governed by the control coordinate *s*, which is the dihedral angle in our DMM study, for example. The control coordinate changes along the dynamical trajectory and generates the PES as a function of s. REG then ranks atomic energies by comparing the ratio of their gradients with that of the total energy. This activity allows one to detect, by (unbiased) computation, which IQA terms behave most like the total molecular energy. Loosely speaking, this means that one can compute which local part of the molecule is in control of its behaviour and why.

The energy profile is separated into segments that are defined by the turning points (maximum and minimum critical points) in the total energy. REG analyses each segment separately, and relates the ratio of the gradient (denoted $m_{{\mathrm{REG}}_{i}}$, which is dimensionless) of an individual energy contribution (denoted $E_{i}\left(s\right)$) to the total energy (gradient) of the system (denoted $E_{\mathrm{total}}\left(s\right)$) by linear regression:

(5) $$E_{i}\left(s\right)=m_{{\mathrm{REG}}_{i}}.E_{\mathrm{total}}\left(s\right)+c_{i}$$

where $m_{{\mathrm{REG}}_{i}}$ (or the “REG value”) is calculated by ordinary least squares regression and $c_{i}$ is the intercept. Note that there is an equation similar to Equation (5) for each energy term i, where the index refers to both the type of energy (e.g., electrostatic) and the locality (e.g., an oxygen atom). The absolute value of the Pearson correlation coefficient (associated with the regression) should be as close as possible to unity. In that case, a REG value emerges from the fit to the energy data points that represent the segment. However, if the Pearson coefficient greatly deviates from unity, then the linear approximation breaks down and the REG value becomes meaningless. Finally, we note that the energies $E_{\mathrm{total}}\left(s\right)$ and $E_{i}\left(s\right)$ are actually shifted by subtracting from them their respective mean energies.

Once the REG values have been calculated, they are ranked from most positive to most negative (i.e., from largest to smallest). A positive REG value means that the gradient of the individual energy contribution acts in the same direction as the total energy over the given segment. In other words, the individual energy contribution behaves similarly to the total energy and greatly helps in constructing its profile. The opposite is true for a negative REG value; in this case, the energy contribution works very much against the total energy profile. The REG method ranks all individual energy terms such that the terms with the largest magnitude REG values are more chemically relevant than IQA terms with smaller magnitude REG values. Due to its exhaustive nature, the REG method detects all effects, no matter how subtle, and ranks them quantitatively.

## 3. Computational Details and Materials

**Figure 1:**
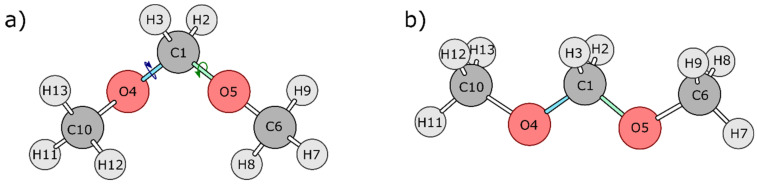
Optimized (**a**) gg and (**b**) tt conformations of DMM.

**Figure 2:**
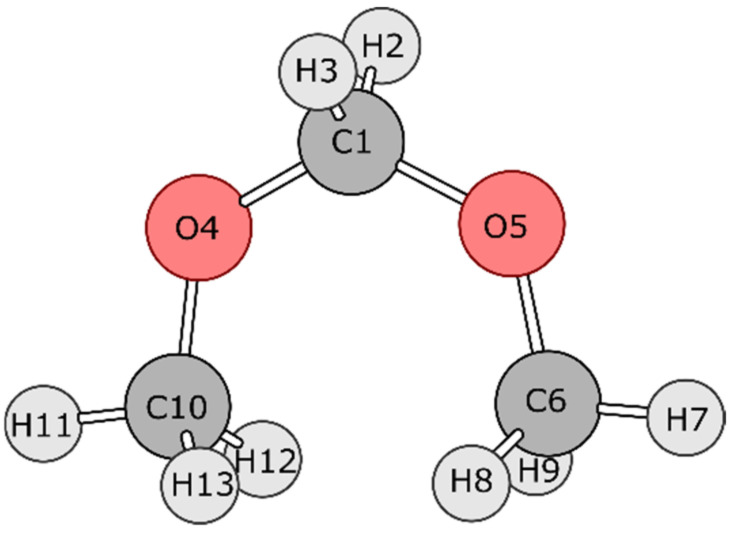
Optimized initial conformation of DMM with both dihedral angles at 0 degrees. The C10 methyl group pops out of the screen while the C6 group goes into the screen as the two associated dihedral angles (O4-C1-O5-C6 and O5-C1-O4-C10) are changed.

Figure 1 shows two relevant conformations of DMM: gauche-gauche (gg) and trans–trans (tt). Through a combined IQA and REG treatment, a dynamical trajectory was generated that allows transitioning between both conformations over a single PES. To do this, we are using only one control coordinate, but one that accounts for simultaneous change in both dihedral angles O4-C1-O5-C6 and O5-C1-O4-C10. Thus, while we monitor O4-C1-O5-C6 as the control coordinate, for example, we know that the other dihedral angle O5-C1-O4-C10 changes simultaneously in order to transition over the conformations (from 0° to 180°) of both terminal methyl groups. Both of these dihedral angles were fixed at 0° in the initial conformation (Figure 2), and then these two dihedral angles were changed simultaneously by 10°, each in opposite directions to generate the other conformations. This procedure allowed the two terminal methyl groups to simultaneously perform rotations about their respective oxygen atoms in directions opposite to each other. The configuration with dihedral angles (70°, −70°) corresponds to the gg conformation while the (180°, −180°) configuration corresponds to the tt conformation. The geometries thus obtained at each increment of 10° dihedral angle (19 structures in total) were optimized at the B3LYP/6-311++G(d,p) level of theory while keeping the O4-C1-O5-C6 and O5-C1-O4-C10 dihedral angles fixed and relaxing all other coordinates. The wavefunction obtained for each geometry was then energy-partitioned using IQA, and the PES was analysed using the REG method.

**Figure 3:**
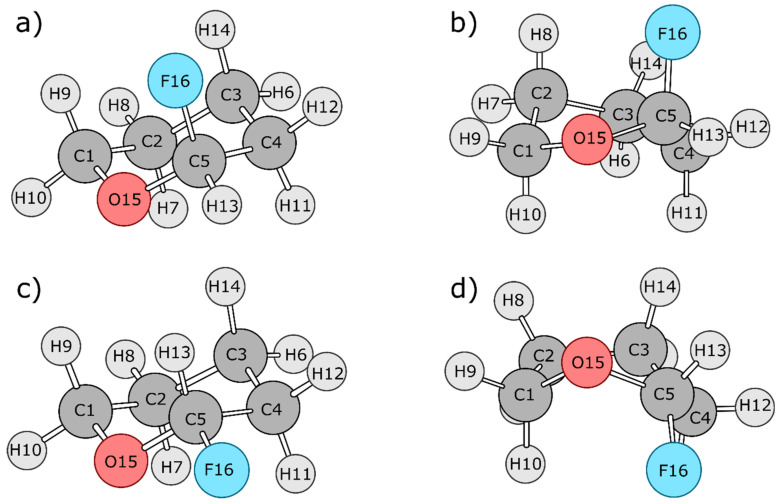
Optimized stereoisomers of FTHP: (**a**) R chair, (**b**) R twist-boat, (**c**) S chair, and (**d**) S twist-boat.

**Figure 4:**
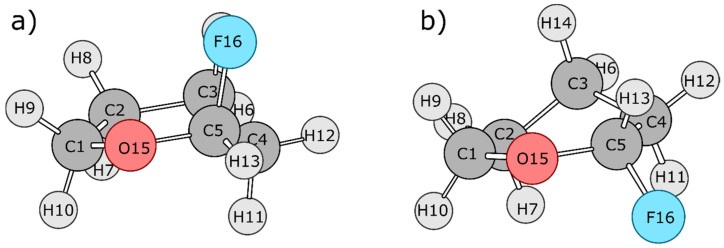
QST-2 optimized transition state geometry for the (**a**) R isomer and (**b**) S isomer of FTHP.

Two separate dynamical trajectories were generated for the analysis of FTHP. In both cases, the molecule transitions from its twist-boat form to its chair form. The two cases are R and S diastereomers with respect to the fluorine substituent (Figure 3), which is attached to the chiral centre C5. The chair form of the R isomer corresponds to the structure in which fluorine is in the axial position; it has the lowest energy due to the anomeric effect. In the first dynamical trajectory, the system transitions from the twist-boat form of the R stereoisomer to the chair form of the R stereoisomer; this transition involves the anomeric effect. In the second dynamical trajectory, the system transitions from the twist-boat form of the S stereoisomer to the chair form of the S stereoisomer; this transition does not involve the anomeric effect. Hence, the difference in the energetic terms between these two transitions aids in spotting the energy contributions leading to the anomeric effect. Figure 4 shows the transition state geometries between the twist-boat and chair conformations for both the systems, which were estimated using the Synchronous Transit-Guided Quasi-Newton (QST-2) method [34].

Intrinsic Reaction Coordinate (IRC) [35] calculations were then run in both forward and backward directions from the QST2-optimized transition state geometry. Single-point energy calculations were run at each point of the IRC path in order to generate the wavefunction files. The wavefunction obtained for each point (i.e., geometry) was then partitioned using the IQA energy partitioning scheme, and the profile of potential energy versus IRC was then analysed using the REG method.

All wavefunctions were obtained through the program [36] GAUSSIAN16, using the B3LYP [37,38] functional in conjunction with the 6-311++G(d,p) basis set. All IQA calculations were performed using the program [39] AIMAll version 16.01.09, while all REG calculations were performed using the in-house program [40] REG.py, written in the Python 3 programming language.

## 4. Results and Discussion

### 4.1. Dimethoxymethane (DMM)

**Figure 5:**
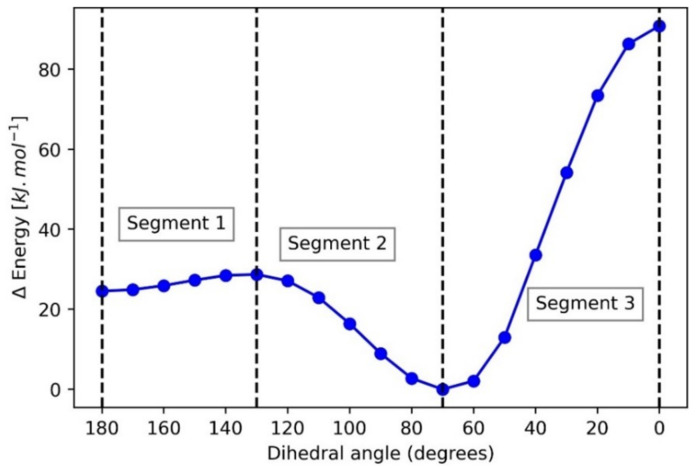
Potential energy surface for DMM as a function of one of the dihedral angles: O5-C1-O4-C10, where tt occurs at 180° and gg at 70°. The dashed vertical lines mark the stationary points that divide the energy profile into 3 segments. An REG analysis is performed for each segment.

The discussion starts with the energy profile of DMM shown in Figure 5. The global minimum for this curve occurs at 70° (i.e., both dihedral angles at 70°), which corresponds to the gg conformation, while the 180° point (i.e., both dihedral angles 180°) corresponds to the tt conformation. Hence, this profile allows a direct comparison between the gg and tt conformations, the difference between which is governed by the generalized (because acyclic) anomeric effect in DMM. The gg conformation is 24.5 kJ∙mol^−1^ more stable than the tt conformation. The four stationary points at 0°, 70°, 130°, and 180° divide the energy profile into three segments. A REG analysis is performed over each segment.

Each segment is bounded (and thus defined) by four stationary points on the PES: segment 1 between 180° and 130°, segment 2 between 130° and 70°, and segment 3 between 70° and 0°. The energy difference between the gg and tt conformations is defined as the anomeric effect in DMM, the transition between which takes place across segments 1 and 2. Hence, these two segments are important. As a quick aside, we explain here why the energy difference between gg and tt naturally corresponds to the anomeric effect. That gg is more stable than tt is the main “surprise” that the anomeric effect introduces, as it is typically illustrated with a cyclohexane containing a heteroatom (e.g., O) and substituted with a substituent also containing a heteroatom (e.g., OCH_3_). The aforementioned surprise comes from the naive intuition, based on steric grounds only, that the open, uncongested conformation (tt) would be lower in energy than the congested one (gg). From Figure 5, it is clear that it is the other way around, and because this result is counterintuitive (naively speaking) the effect has a name: the anomeric effect.

Table 1 shows the energy terms with the largest magnitude REG values for each of the three segments. The Supplementary Materials contains the data (energy type, localization, REG value, and R) for all the energy terms. Table 1 and similar tables below contain a relevant subsection of these data.

Here, we analyse segment 1 first. Moving away from the local minimum at tt (180°) towards the transition state at 130° increases the total energy and thus destabilizes the whole system. Thus, energy terms with negative REG values do the opposite: they decrease in energy and hence stabilize. The energy term that stabilizes the most across segment 1 is the intra-atomic energy of the central carbon atom, E_intra_(C1), displaying the most negative REG value of −13.52. This indicates large stabilizing changes in the internal electron density of the central carbon atom. Next, there is the ∑V_cl_(O,H) term, with the second most negative REG value of −7.80. This energy term is a summation of the four attractive 1,3 electrostatic interaction terms between the central hydrogen atoms (H2 and H3) and the two oxygen atoms (i.e., V_cl_(H2,O4), V_cl_(H2,O5), V_cl_(H3,O4), and V_cl_(H3,O5)). Because these interactions are symmetric and have the same REG values (up to 2 decimal places) across all segments, they have been summed up in order to analyse them as a single energy term. The negative REG value again means that this (collective) electrostatic interaction stabilizes the system en route to the transition state. Finally, the positive REG values also add to the explanation of this energy barrier (when moving from left to right). Because they are positive, they help create the barrier and are thus destabilizing. Indeed, the two central (ideally symmetric) 1,2 C-O electrostatic interaction energies have large REG values (11.8 and 11.3) and destabilize the system most, indicating increased electrostatic repulsion between the central carbon and the two oxygen atoms.

Moving on to segment 2, we see that the ∑V_cl_(O,H) term is the most important as it shows the largest positive REG value. Next appears carbon-oxygen electrostatic interactions, now of 1,4 nature and thus involving the methyl carbons, followed by the intra-atomic energies of the three carbon atoms. The E_intra_(C1) term noted in the previous segment appears here only as the 6th most stabilizing energy term. However, it has a poor Pearson correlation coefficient value of 0.72 across this segment, which means that it cannot be judged properly through REG alone. An important observation to be made through the REG analysis is the lack of *exchange-correlation terms across segments 1 and 2, indicating that these effects do not have a significant role.* It is tempting to regard the V_xc_(C1,O5) and V_xc_(C1,O4) terms as representing the textbook hyperconjugation interaction between the oxygen lone pair and the C1 carbon atom. These interactions have small REG values of only 0.60 and 0.16 across segments 1 and 2, respectively, and hence did not make it into Table 1. In summary, the anomeric effect is dominated by electrostatic and steric effects, as can be seen from their REG values across segments 1 and 2.

Table 2 contains the energy terms with the largest magnitude of IQA energy *difference* between the gg and tt conformations. The energy terms with negative values stabilize the gg conformation over the tt conformation and vice versa. The two energy terms with the most negative ∆E(gg − tt) values are E_intra_(C1) and ∑V_cl_(H,O), or the 1,3 electrostatic interactions between the central hydrogens and the two oxygens. Highlighted by the REG analysis just above, they confirm the insight gained from it. These two energy terms can be designated as the contributions most responsible for the anomeric effect in DMM. Likewise, the energy terms that destabilize the gg conformation the most (50.1) are the two 1,2 C-O electrostatic interactions between the central carbon (C1) and the two oxygen atoms. Moreover, and importantly, the repeated absence in Table 2 of substantial exchange-correlation effects confirms again that hyperconjugation does not play much of a role.

**Figure 6:**
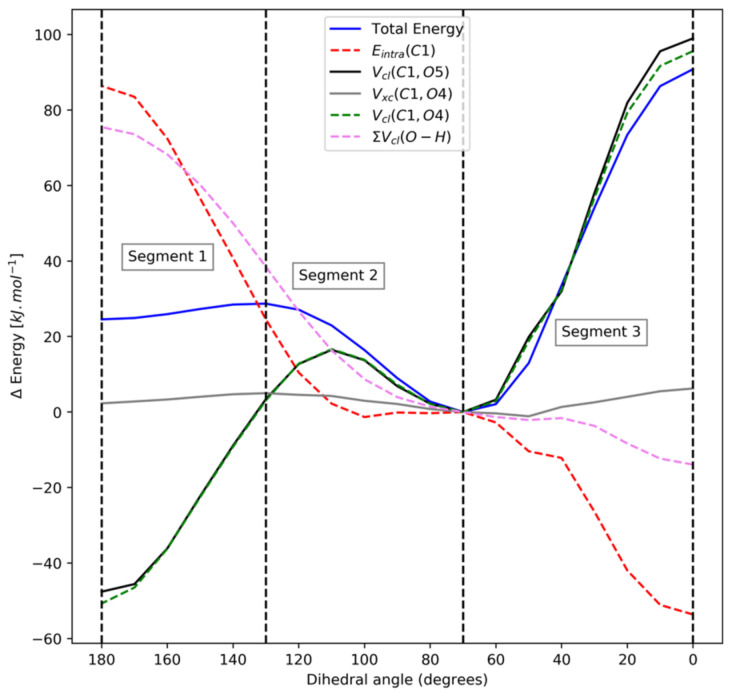
Individual and total energy profiles of the most important contributors over the 3 segments in DMM supplemented with V_xc_(C1,O4).

Figure 6 shows the individual and total energy profiles of the most important contributors over the three segments in DMM supplemented with V_xc_(C1,O4). Analysing Figure 6 helps to confirm the insights gained from the REG analysis and also helps in judging energy terms with poor Pearson correlation coefficient values. A striking observation is the behaviour of the V_xc_(C1,O4) term, associated with hyperconjugation, turns out to be almost constant over the whole angular variation. This finding drives home the point that no hyperconjugation effects are at play here. Figure 6 shows the almost symmetric and simultaneous stabilization and destabilization of the E_intra_(C1) and V_cl_(C1,O4)|V_cl_(C1,O5) energy terms, respectively, and large monotonic change over the whole angular interval. This observation is interesting because we believe that it may point towards charge transfer, which can be analysed through QTAIM charges shown in Table 3.

Table 3 shows that the positive net charge on C1 decreases as the system transitions from the tt to the gg structure. Thus, C1 gains (negative) electronic charge as the global minimum (gg) is reached. On the other hand, the hydrogen atoms attached to C1 (H2 and H3) lose electronic charge. The electronic charge gained by C1 is the largest in magnitude out of all atoms, while H2 and H3 lose the largest magnitude of electronic charge of all. The changes in the oxygen values are about three times smaller compared to those of both the hydrogens and C1 (by absolute value). The oxygens also gain electronic charge while going to gg. If charge–charge interaction dominates the full electrostatic interaction, then ∑V_cl_(H,O) becomes more and more negative toward gg because O becomes more negative while H becomes more positive. Thus, ∑V_cl_(H,O) becomes more attractive, which is consistent with its stabilizing nature during the transition from tt to gg (over segment 1 and 2). By similar logic, the electrostatic interaction between C1 and O5 (or between C1 and O4) is also affected by a double gain of electronic charge (i.e., for C and O) going from tt to gg. Thus, more and more repulsion arises in the attractive V_cl_(C1,O4), which leads to destabilization. Again, this is consistent with what is seen in Figure 6 and with the REG analysis. Finally, this charge transfer also leads to a stabilization of the intra-atomic energy of C1. The partitioning of intra-atomic energy contribution, under the IQA framework, into steric hindrance and charge transfer components has recently been shown [41] by Gallegos et al. This charge transfer in DMM has previously been noted by Vila and Mosquera [41]. No hyperconjugation is at play here.

### 4.2. 2-Fluorotetrahydropyran (FTHP)

**Figure 7:**
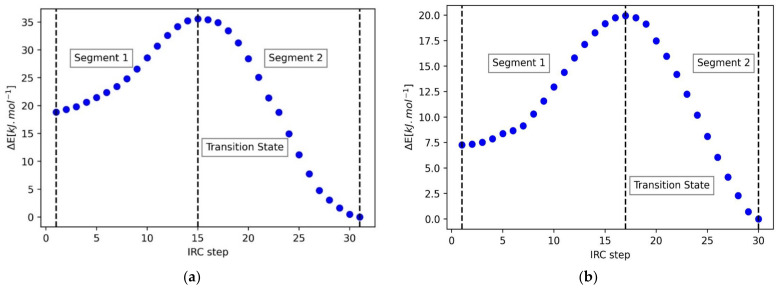
IRC path for (**a**) the R isomer, in the presence of the anomeric effect, and (**b**) the S isomer, in the absence of the anomeric effect. The initial and final geometries correspond to the twist-boat and chair forms, respectively, for both isomers.

We now study the 2-fluorotetrahydropyran (FTHP) molecule in order to verify whether the trends and mechanism noted from the DMM case study carry over to a cyclic model of the anomeric effect. Figure 7 shows the PES obtained through IRC calculations for the two isomers, R and S. The initial and final geometries correspond to the twist-boat and chair forms, respectively, for both isomers. In the R isomer, the chair is 18.7 kJ∙mol^−1^ more stable than the twist-boat, but in the S isomer, the chair is only 4.9 kJ∙mol^−1^ more stable. This reduced magnitude of energy difference in the S isomer indicates the absence of the anomeric effect in this system.

As before, we now carry out a REG analysis on the PES in Figure 7 across the two segments for both isomers. The three stationary points (initial geometry, transition state, and final geometry) of the PES divide the curve into two segments. Table 4 shows the energy terms with the largest magnitude REG values for both segments in the *R* isomer. Note that some energy terms corresponding only to the changes in the ring structure have not been included in our analysis since these are not relevant to our study.

The total energy in segment 1 increases, so energy terms with negative REG values stabilize the system as it transitions away from the twist-boat. The 1,2-type V_cl_(C5,O15) term stabilizes the most (−3.69), but remarkably, this term also destabilizes the most in segment 2. The next two terms that stabilize the most in segment 1 are E_intra_(C1) (C1 is adjacent to O15) and the 1,3-type V_cl_(H10,O15) (H10 is bonded to C1). On the other hand, the term that destabilizes the most in segment 1 is the 1,2-type V_cl_(C1,O15). This interplay between the C1, H10, and O15 atoms is similar to the mechanism observed in DMM between the E_intra_(C1), ∑V_cl_(H,O), and V_cl_(C1,O4)/V_cl_(C1,O5) terms. 

Another important similarity with the DMM case is that, once again, the term corresponding to the hyperconjugation interaction between the oxygen lone pair and C-X sigma* bond (V_xc_(C5,O15)) has very small absolute REG values across both segments for the R isomer. However, this term has unacceptably small Pearson correlation coefficient values across both segments, so the REG values are not accurate. Furthermore, this contribution has a ∆E (chair minus twist-boat) value of only –3.7 kJ∙mol^−1^, which indicates that hyperconjugation is not responsible for the anomeric effect in FTHP either.

Looking at segment 2 reveals that the two terms that stabilize the most are the intra-atomic energies of the C5 and O15 atoms, with the largest positive REG values. However, these two terms also destabilize greatly in segment 1, with large positive REG values. Hence, one expects their overall effect to cancel out. Apart from these terms, several of the other energy terms have poor Pearson correlation coefficient values across segment 2, so these cannot be judged properly through REG alone. We reserve judgment after the analysis of the IQA energy differences and QTAIM charges.

We now move on to the REG analysis for the S isomer shown in Table 5, which is the equivalent of Table 4. Because the anomeric effect is absent in this geometry, the mechanism noted in DMM and that in the R isomer should also be absent here. 

Because the total energy increases in segment 1, negative REG values correspond to stabilization and vice versa. The term with the largest positive REG value in segment 1 is E_intra_(C1), indicating that this contribution destabilizes the most. Meanwhile, the 1,2-type V_cl_(C1,O15) energy term stabilizes across both segments, with negative and positive REG values across segments 1 and 2, respectively. These two observations are exactly opposite to the trend noted in both DMM and the R isomer. Because we are only interested in the differences between the two isomers and not in the stability of the S isomer, we end this discussion by briefly noting that the E_intra_(O15) term and the 1,2-type V_cl_(C5,F16) term, with highly negative and positive REG values in segments 1 and 2, respectively, are the most stabilizing energy contributions for the chair conformation in the S isomer. 

**Figure 8:**
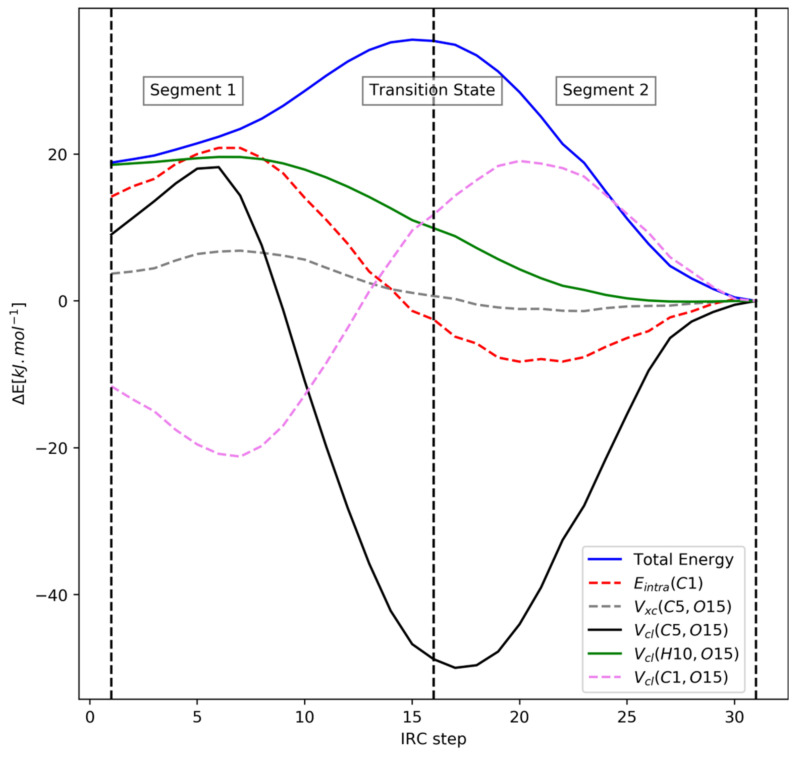
Visualization of the energetic terms in the R isomer.

We now look at the IQA energy difference analysis presented in Table 6, and at Figure 8, which shows the trajectory of important energy terms for the R isomer spotted through the REG analysis. These two analyses will help us confirm the insights gained from the preceding REG analysis and also allow us to better judge energetic terms with poor Pearson correlation coefficients in Table 4.

**Figure 9:**
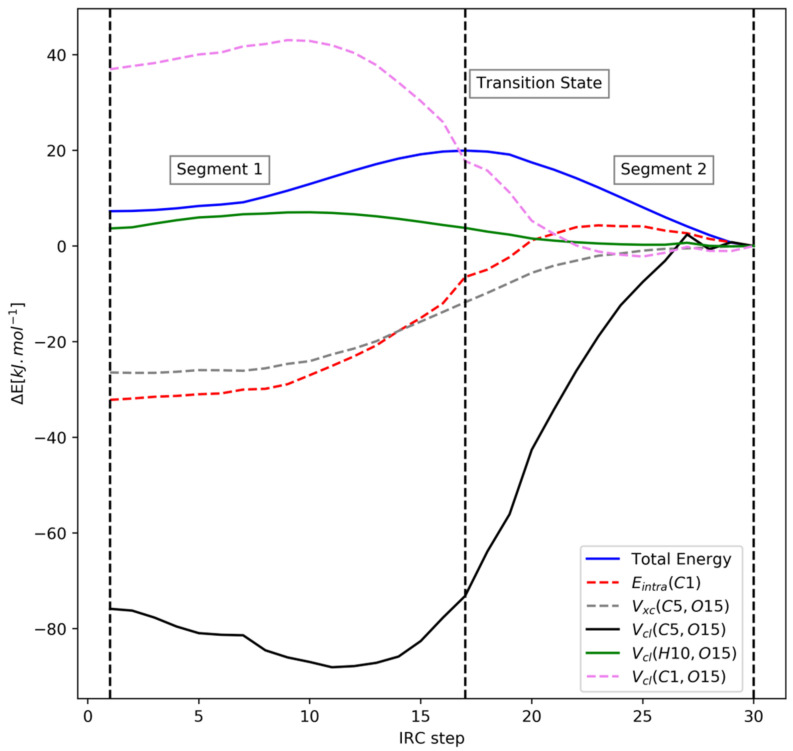
Visualization of the same energetic terms (of the R isomer) in the S isomer.

In Table 6, the energy terms with negative values stabilize the chair form over the twist-boat form and vice versa. The two most stabilizing energetic terms for the R isomer are the attractive electrostatic interaction term V_cl_(H10,O15) and the intra-atomic energy of C1. This is in line with the observation made from the REG analysis, and these two energetic terms are analogous to the E_intra_(C1) and ∑V_cl_(H-O) terms highlighted in DMM. Another similarity with the DMM case can be seen in Figure 9, where we note the symmetry between the E_intra_(C1) and the V_cl_(C1,O15) energy curves. This is similar to the symmetry between the E_intra_(C1) and V_cl_(C1,O4)/V_cl_(C1,O5) energy curves in DMM noted earlier in Figure 6. Furthermore, the stabilizing V_cl_(H10,O15) is missing in the S isomer and the E_intra_(C1) term instead destabilizes with a ∆E (chair minus twist-boat) value of 32.2 kJ∙mol^−1^, which also indicates that these two terms are related to the anomeric effect.

Secondly, a general lack of (important) exchange-correlation terms in Table 6 once again shows that hyperconjugation effects are not at play in either isomer. Figure 8 shows that the V_xc_(C5,O15) contribution corresponding to C5-O15 hyperconjugation remains almost constant during the transition.

We now look at the QTAIM charge analysis for both isomers in Table 7 to see whether a charge transfer mechanism similar to the DMM case is at play in the R isomer as well.

The QTAIM charge analysis in Table 7 shows that the C1 atom gains electrons in the R isomer, which leads to the stabilization of its intra-atomic energy E_intra_(C1), while H10 (bonded to C1) loses electronic charge, leading to stabilization of the attractive V_cl_(H10,O15) interaction (as H10 becomes more positive and O15 barely changes). This is further supported by the observation that the V_cl_C1,O15) and V_cl_(C1,H10) interactions destabilize during the transition. This charge transfer is missing in the case of the S isomer, where the C1 and C5 atoms lose electrons, and thus, both the E_intra_(C1) and E_intra_(C5) terms destabilize greatly. Similar to these observations, Wang et al. noted [10] a significant charge shift from the hydrogen atoms to the carbon atom adjacent to the ring heteroatom (C1), which leads to enhanced Coulombic interaction between these equatorial hydrogen atoms and the ring heteroatom. This enhanced hydrogen-oxygen Coulombic interaction was also noted [11] by Wiberg et al., but both studies failed to spot the stabilization of the E_intra_(C1) term that we have noted here. Finally, we note that the (absolute) change in charges on C5, O15, F16, and C1 in the S isomer are about three to six times larger than in the R isomers. This disparity may explain why the extreme (electrostatic) energy contributions in the S isomer (V_cl_(H10,O15) and V_cl_(C1,H10)) are several times larger than their counterparts in the R isomer (V_cl_(C5,F16) and V_cl_(C5,O15)).

## 5. Conclusions

The anomeric effect has been studied on both dimethoxymethane (DMM) (which is acyclic) and 2-fluorotetrahydropyran (FTHP) (which is cyclic and has the R and S isomer). The Relative Energy Gradient (REG) method was used, which essentially calculates chemical interpretations in a minimal way. REG operates on Interacting Quantum Atoms (IQA) energies, which cover both intra-atomic and interatomic energy contributions, with the latter being of the electrostatic and exchange(-correlation) type. This analysis was supplemented with QTAIM (atomic) charges. 

An electronic charge shift takes place, both in DMM and FTHP, when moving from a higher-energy geometry to a lower-energy geometry, which is stabilized by the anomeric effect. Electronic charge migrates to the central carbon in DMM while the hydrogen atoms attached to it lose electronic charge. In the R isomer of FTHP, the non-substituted carbon adjacent to the ring’s oxygen also gains electronic charge while a hydrogen attached to the carbon loses electronic charge. This charge transfer leads to (a) stabilization of the intra-atomic energy of the carbon atom, and (b) increased Coulombic attraction between the hydrogen atoms and the heteroatoms in both DMM and FTHP. These two energetic contributions are primarily responsible for the anomeric effect in both molecules. Our study also shows the absence of any significant stabilization due to exchange(-correlation) effects in either molecule, which conclusively shows that the hitherto put forward hyperconjugation mechanism is invalid.

## Figures and Tables

**Table 1:** Largest magnitude REG values across the three energy segments of DMM along with their Pearson correlation coefficient (R) values. Unacceptably low R values are marked in bold.

Segment 1			Segment 2			Segment 3		
Contribution	REG	R	Contribution	REG	R	Contribution	REG	R
V_cl_(C1,O4)	11.77	0.99	∑V_cl_(O,H)	1.14	**0.92 ± 0.01**	V_cl_(C1,O5)	1.08	1.00
V_cl_(C1,O5)	11.34	0.98	V_cl_(O4,C6)	0.79	0.99	V_cl_(C1,O4)	1.05	1.00
V_cl_(C1,H2)	3.22	0.96	V_cl_(O5,C10)	0.79	0.99	E_intra_(C10)	0.50	0.97
V_cl_(C1,H3)	3.22	0.96	E_intra_(C10)	0.71	0.99	E_intra_(C6)	0.50	0.97
V_cl_(O5,C6)	2.92	0.99	E_intra_(C6)	0.70	0.99	V_xc_(O4,O5)	0.22	0.99
V_cl_(O4,C10)	2.91	0.99	E_intra_(C1)	0.58	0.72	V_cl_(C6,C10)	0.20	0.95
V_cl_(H2,O4)	−1.93	−0.96	V_cl_(C1,O5)	0.33	0.60	V_cl_(C1,C6)	0.20	−0.96
V_cl_(H3,O5)	−1.93	−0.96	V_cl_(O5,H8)	0.33	1.00	∑V_cl_(O,H)	−0.13	−0.93 ± 0.02
V_cl_(H3,O4)	−1.97	−0.96	V_cl_(C6,C10)	−0.39	−0.97	E_intra_(O4)	−0.30	−0.91
V_cl_(H2,O5)	−1.97	−0.96	V_cl_(C1,H2)	−0.45	−0.92	E_intra_(O5)	−0.33	−0.93
E_intra_(O5)	−4.48	−0.99	V_cl_(C1,H3)	−0.45	−0.92	V_cl_(O4,O5)	−0.36	−0.99
E_intra_(O4)	−4.70	−0.99	V_cl_(O4,O5)	−0.55	−1.00	V_cl_(O5,C6)	−0.56	−0.89
∑V_cl_(O,H)	−7.80	−0.96 ± 0.00	V_cl_(O5,C6)	−0.66	−0.98	V_cl_(O4,C10)	−0.57	−0.89
E_intra_(C1)	−13.52	−0.98	V_cl_(O4,C10)	−0.67	−0.98	E_intra_(C1)	−0.57	−0.99

**Table 2:** A few of the largest-magnitude IQA energy differences between gg and tt.

Contribution	∆E(gg − tt) (kJ∙mol^−1^)
E_intra_(C1)	−87.9
∑V_cl_(H,O)	−75.6
V_cl_(O4,C6)	−29.6
V_cl_(O5,C10)	−29.3
V_cl_(O5,C6)	34.3
V_cl_(O4,C10)	34.8
V_cl_(C1,O5)	50.1
V_cl_(C1,O4)	50.1

**Table 3:** QTAIM charges (in electron, e) of all atoms in DMM for its gg and tt conformers, and their difference.

Atoms	Q_tt_	Q_gg_	∆Q_gg − tt_
C1	1.0312	0.9927	−0.0385
H2	−0.0086	0.0225	0.0311
H3	−0.0086	0.0225	0.0311
O4	−1.0358	−1.0454	−0.0095
O5	−1.0353	−1.0456	−0.0102
C6	0.4962	0.4813	−0.0149
H7	0.0351	0.0262	−0.0089
H8	−0.0015	0.0196	0.0211
H9	−0.0015	−0.0004	0.0011
C10	0.4960	0.4811	−0.0148
H11	0.0352	0.0262	−0.0090
H12	−0.0015	0.0195	0.0209
H13	−0.0015	−0.0004	0.0010

**Table 4:** REG analysis for the R isomer. Unacceptably low R values are marked in bold.

Segment 1			Segment 2		
Contribution	REG	R	Contribution	REG	R
V_cl_(C1,O15)	1.33	0.82	E_intra_(C5)	0.62	0.99
E_intra_(C5)	1.29	0.99	E_intra_(O15)	0.57	0.97
E_intra_(O15)	1.18	0.97	V_cl_(C1,O15)	0.37	**0.76**
V_cl_(C5,F16)	0.46	**0.61**	V_cl_(C1,F16)	0.33	0.97
V_cl_(C1,H10)	0.37	0.91	V_cl_(H10,O15)	0.26	0.91
V_cl_(O15,F16)	0.37	0.99	V_cl_(C5,F16)	0.20	**0.65**
V_xc_(C5,F16)	0.36	0.92	V_xc_(C5,O15)	0.01	**0.16**
V_xc_(C5,O15)	−0.19	**−0.63**	V_cl_(H14,O15)	−0.11	−0.96
E_intra_(F16)	−0.26	**−0.58**	E_intra_(C1)	−0.12	**−0.54**
V_xc_(C1,H10)	−0.39	−0.99	E_intra_(F16)	−0.20	−0.80
V_cl_(H10,O15)	−0.39	−0.88	V_cl_(C1,H10)	−0.25	−0.94
E_intra_(C1)	−1.00	−0.85	V_cl_(C1,C5)	−0.31	−0.88
V_cl_(C5,O15)	−3.69	−0.96	V_cl_(C5,O15)	−1.47	−0.99

**Table 5:** REG analysis for the S isomer. Unacceptably low R values are marked in bold.

Segment 1			Segment 2		
Contribution	REG	R	Contribution	REG	R
E_intra_(C1)	1.56	0.95	V_cl_(C5,F16)	3.73	0.99
E_intra_(C5)	1.45	0.99	E_intra_(O15)	1.06	0.93
V_xc_(C5,O15)	0.96	0.95	V_xc_(C1,O15)	0.78	0.98
V_cl_(C1,F16)	0.94	0.94	V_cl_(C1,O15)	0.70	**0.75**
V_cl_(H9,O15)	0.73	0.97	V_cl_(C5,H13)	0.66	0.97
V_xc_(C1,O15)	0.66	0.97	V_xc_(C5,F16)	0.62	0.99
V_cl_(H13,O15)	0.48	0.94	E_intra_(H13)	0.35	0.97
V_xc_(H14,O15)	0.40	1.00	V_cl_(C5,H9)	0.30	0.91
V_xc_(O15,F16)	−0.47	−0.94	V_xc_(C5,H13)	−0.28	−1.00
V_cl_(C5,H9)	−0.62	−0.98	V_cl_(H9,O15)	−0.32	−0.88
V_cl_(C5,F16)	−0.71	**−0.55**	V_xc_(C5,O15)	−0.47	−0.88
V_cl_(C1,H9)	−0.75	−0.99	V_cl_(H13,O15)	−0.51	−0.97
V_cl_(C5,H13)	−0.78	−0.96	V_cl_(C1,C5)	−0.59	−0.99
V_cl_(C1,O15)	−0.91	**−0.65**	E_intra_(C5)	−1.27	−0.97
V_cl_(C1,C5)	−1.03	−0.88	E_intra_(F16)	−1.39	−0.95
E_intra_(O15)	−1.11	−0.99	V_cl_(C5,O15)	−3.34	−0.93

**Table 6:** A few of the largest-magnitude IQA energy differences (kJ∙mol^−1^) for the R and S isomers.

R Isomer		S Isomer	
Contribution	∆E (Chair Minus Twist-Boat)	Contribution	∆E (Chair Minus Twist-Boat)
V_cl_(H10,O15)	−18.53	V_cl_(C5,F16)	−96.47
E_intra_(C1)	−14.20	E_intra_(O15)	−39.15
V_cl_(C5,O15)	−9.03	V_cl_(C1,O15)	−36.96
V_cl_(C1,F16)	−7.80	V_cl_(C5,H13)	−27.14
V_cl_(H10,F16)	−7.39	V_cl_(C1,H9)	−18.34
V_xc_(C1,C4)	7.18	E_intra_(C1)	32.17
V_cl_(C1,O15)	11.61	E_intra_(F16)	36.07
V_cl_(C5,H10)	14.23	E_intra_(C5)	48.22
V_cl_(C1,H10)	17.24	V_cl_(C5,O15)	75.86

**Table 7:** QTAIM charges (in electron, e) for the R and S isomers.

	R Isomer			S Isomer		
Atoms	Twist-Boat	Chair	Chair-Twist-Boat	Twist-Boat	Chair	Chair-Twist-Boat
C1	0.4649	0.4557	−0.0092	0.4540	0.4778	0.0238
C2	0.0431	0.0461	0.0030	0.0393	0.0455	0.0062
C3	0.0488	0.0506	0.0019	0.0592	0.0472	−0.0120
C4	0.0485	0.0518	0.0033	0.0406	0.0444	0.0038
C5	0.9782	0.9811	0.0029	0.9865	1.0053	0.0187
H6	−0.0125	−0.0094	0.0031	−0.0065	−0.0037	0.0028
H7	−0.0057	−0.0125	−0.0068	−0.0117	−0.0021	0.0096
H8	0.0103	−0.0048	−0.0151	−0.0071	−0.0062	0.0009
H9	0.0274	0.0177	−0.0096	0.0235	−0.0083	−0.0319
H10	−0.0053	0.0242	0.0295	0.0219	0.0289	0.0070
H11	−0.0033	−0.0010	0.0023	0.0201	0.0164	−0.0038
H12	0.0141	0.0098	−0.0043	−0.0002	0.0043	0.0045
H13	0.0466	0.0510	0.0044	0.0504	0.0226	−0.0278
H14	0.0069	0.0052	−0.0016	−0.0056	−0.0197	−0.0141
O15	−1.0438	−1.0462	−0.0025	−1.0454	−1.0344	0.0110
F16	−0.6184	−0.6191	−0.0008	−0.6192	−0.6171	0.0022

## Data Availability

Any data generated and analysed for this study that are not included in this communication are available from the authors upon reasonable request.

## Supplementary Materials

The following supporting information can be downloaded at: https://www.mdpi.com/article/10.3390/molecules27155003/s1, Table S1. REG values of all interactions in DMM; Table S2. REG values of all interactions in the FTHP-R isomer; Table S3. REG values of all interactions in the FTHP-S isomer.

## Sample Availability

Data are available from the authors upon request.
