# Supplementary material for: An Interacting Quantum Atoms (IQA) and Relative Energy Gradient (REG) Analysis of the Anomeric Effect
Source: Molecules. 2022 Aug 6;27(15):5003. doi: 10.3390/molecules27155003 (PMC9370807; doi:10.3390/molecules27155003)
Supplement: Supplementary file 1 [file molecules-27-05003-s001.zip › molecules-1836207-supplementary.pdf]

Supporting Information

An Interacting Quantum Atoms (IQA) and

Relative Energy Gradient (REG) Analysis of the Anomeric Effect

Danish Khan, Leonardo J. Duarte and Paul L. A. Popelier*

Content:

Table S1. REG values of all interactions in DMM.

Table S2. REG values of all interactions in the FTHP-R isomer.

Table S3. REG values of all interactions in the FTHP-S isomer.

Table S1. REG values of all interactions in DMM.

**Segment 1**			**Segment 2**			**Segment 3**		
**Contribution**	**REG**	**R**	**Contribution**	**REG**	**R**	**Contribution**	**REG**	**R**
VCl-C1,O4	11.77	0.99	VCl-O4,C6	0.79	0.99	VCl-C1,O5	1.08	1.00
VCl-C1,O5	11.34	0.98	VCl-O5,C10	0.79	0.99	VCl-C1,O4	1.05	1.00
VCl-C1,H2	3.22	0.96	Eintra-C10	0.71	0.99	Eintra-C10	0.50	0.97
VCl-C1,H3	3.22	0.96	Eintra-C6	0.70	0.99	Eintra-C6	0.50	0.97
VCl-O5,C6	2.92	0.99	Eintra-C1	0.58	0.72	VxC-O4,O5	0.22	0.99
VCl-O4,C10	2.91	0.99	VCl-C1,O5	0.33	0.60	VCl-C6,C10	0.20	0.95
Eintra-H3	1.16	0.99	VCl-O5,H8	0.33	1.00	VCl-O5,H8	0.14	0.92
Eintra-H2	1.16	0.99	VCl-O4,H12	0.33	1.00	VCl-O4,H12	0.13	0.91
VxC-O5,C6	1.04	0.97	VCl-C1,O4	0.33	0.58	VxC-O5,H12	0.10	0.88
VxC-O4,C10	1.02	0.97	VCl-H2,O5	0.29	0.93	Eintra-H3	0.09	0.99
VxC-H3,H9	0.96	0.96	VCl-H3,O4	0.29	0.93	Eintra-H2	0.09	0.99
VxC-H2,H13	0.96	0.96	VCl-H3,O5	0.28	0.92	VCl-O4,H8	0.09	0.84
VCl-H3,C6	0.92	0.97	VCl-H2,O4	0.28	0.92	VxC-O4,H8	0.09	0.75
VCl-H2,C10	0.92	0.97	VxC-O4,H8	0.27	0.95	Eintra-H13	0.08	0.89
VCl-H3,C10	0.85	0.96	VxC-O5,H12	0.27	0.95	Eintra-H9	0.08	0.90
VCl-H2,C6	0.85	0.96	VCl-O4,H8	0.23	0.99	VxC-C1,O5	0.08	0.98
VCl-C6,H8	0.67	0.98	VCl-O5,H12	0.23	0.99	VxC-C1,O4	0.07	0.97
VCl-C10,H12	0.66	0.98	VxC-C1,O4	0.16	0.99	VxC-O4,C6	0.07	0.88
VxC-C1,O4	0.60	1.00	VxC-C1,O5	0.16	1.00	VxC-O5,C10	0.07	0.89
VCl-O4,H13	0.57	0.93	VxC-H3,H13	0.14	0.89	VCl-O5,H12	0.06	0.90
VxC-H3,C6	0.57	0.90	VxC-O4,C6	0.14	0.86	VxC-C6,H7	0.05	1.00
VxC-H2,C10	0.57	0.90	VxC-O5,C10	0.14	0.86	VxC-C10,H11	0.05	1.00
VCl-O5,H9	0.56	0.94	VxC-H2,H9	0.14	0.89	VxC-H2,H9	0.05	0.92
VCl-O4,H11	0.56	0.99	VCl-C10,H11	0.12	0.99	VxC-H3,H13	0.05	0.92
VCl-O5,H7	0.56	0.99	VCl-C6,H7	0.12	0.99	VCl-O4,H9	0.05	0.70
VxC-C1,O5	0.54	1.00	VxC-C6,H8	0.09	0.83	VCl-O5,H13	0.05	0.69
Eintra-C10	0.52	0.96	VxC-C10,H12	0.09	0.83	VxC-O4,C10	0.05	0.92
Eintra-C6	0.46	0.97	VCl-O4,H13	0.08	0.98	VxC-O5,C6	0.05	0.92
Eintra-H8	0.37	0.97	VCl-O5,H9	0.08	0.98	VxC-H8,H12	0.04	0.33
Eintra-H12	0.37	0.97	Eintra-H3	0.08	0.76	Eintra-H11	0.04	0.99
VCl-C1,H8	0.35	0.96	Eintra-H2	0.08	0.76	Eintra-H7	0.04	0.99
VCl-C1,H12	0.35	0.96	VxC-C10,H11	0.07	1.00	VCl-C1,H2	0.04	0.92
VxC-C1,H9	0.30	1.00	VxC-C6,H7	0.07	1.00	VxC-C1,C10	0.04	0.96
VxC-C1,H13	0.30	1.00	VCl-O4,H9	0.07	0.98	VxC-C1,C6	0.04	0.96
VxC-H2,H3	0.29	0.97	VCl-O5,H13	0.07	0.98	VCl-C1,H3	0.04	0.92
VxC-C1,C10	0.26	0.99	Eintra-H11	0.05	0.98	VCl-C10,H11	0.04	0.63
VxC-C1,C6	0.26	1.00	Eintra-H7	0.05	0.98	VCl-C6,H7	0.03	0.64
VCl-C6,C10	0.23	0.87	VxC-C1,H13	0.05	0.98	VxC-H3,C10	0.03	0.81
VxC-H3,O4	0.16	0.98	VxC-C1,H9	0.05	0.98	VxC-H2,C6	0.03	0.81
VxC-H2,O5	0.15	0.98	VCl-C1,H11	0.05	0.91	VxC-C1,H9	0.03	0.95
VCl-H2,H3	0.15	0.94	VCl-C1,H7	0.05	0.91	VxC-C1,H13	0.03	0.94
VCl-O5,H11	0.13	1.00	VxC-C1,H2	0.04	0.48	VCl-O5,H9	0.02	0.22
VCl-O4,H7	0.13	1.00	VxC-C1,H3	0.04	0.48	VCl-O4,H13	0.02	0.21
VCl-H8,C10	0.13	0.97	VxC-H3,C6	0.04	0.99	VCl-H7,C10	0.02	0.92

VCl-C6,H12	0.13	0.97	VxC-H2,C10	0.04	0.99	VCl-C6,H11	0.02	0.92
VxC-O4,H12	0.12	0.98	VCl-O5,H11	0.04	0.92	VCl-H2,C10	0.02	0.92
VxC-O5,H8	0.12	0.98	VCl-O4,H7	0.04	0.92	VCl-H3,C6	0.02	0.92
VCl-H3,H7	0.08	0.99	VxC-H3,O5	0.03	0.98	VxC-C10,H12	0.02	0.70
VCl-H2,H11	0.08	0.99	VxC-H2,O4	0.03	0.98	VxC-C6,H8	0.02	0.70
VxC-O5,C10	0.08	0.85	VxC-H8,H12	0.01	0.64	VCl-H3,C10	0.01	0.87
VxC-O4,C6	0.08	0.84	VxC-H8,C10	0.01	0.73	VCl-H2,C6	0.01	0.87
VCl-H2,H9	0.06	0.98	VxC-C6,H12	0.01	0.73	VxC-H3,C6	0.01	0.93
VCl-H3,H13	0.06	0.98	VxC-O5,H11	0.01	0.86	VxC-H2,C10	0.01	0.93
VxC-C10,H11	0.06	0.98	VxC-O4,H7	0.01	0.86	VCl-H2,H3	0.01	0.97
VxC-C6,H7	0.05	0.97	VxC-H2,O5	0.00	0.35	VxC-C1,H12	0.01	0.92
VCl-H3,H11	0.05	0.91	VxC-H3,O4	0.00	0.32	VCl-H9,H12	0.01	0.96
VCl-H2,H7	0.05	0.91	VxC-H3,H7	0.00	0.99	VCl-H8,H13	0.01	0.96
VxC-C6,C10	0.04	0.98	VxC-H2,H11	0.00	0.99	VxC-O5,H11	0.00	0.92
VCl-H7,H8	0.03	0.94	VCl-H2,H13	0.00	0.74	VxC-O4,H7	0.00	0.92
VCl-H11,H12	0.03	0.95	VCl-H3,H9	0.00	0.74	VCl-H7,H11	0.00	0.94
VxC-H7,H8	0.03	0.69	VxC-H8,H13	0.00	1.00	VCl-H2,H7	0.00	0.95
VxC-H11,H12	0.03	0.69	VxC-H9,H12	0.00	1.00	VCl-H3,H11	0.00	0.95
VCl-H3,H8	0.02	0.93	VxC-C6,C10	0.00	0.44	VxC-H11,H13	0.00	0.78
VCl-H2,H12	0.02	0.93	VxC-C6,H13	0.00	0.97	VxC-H7,H9	0.00	0.79
VxC-O5,H7	0.01	0.74	VxC-H9,C10	0.00	0.97	VxC-H2,H11	0.00	0.86
VxC-O5,H11	0.01	0.97	VxC-H7,H12	0.00	0.62	VxC-H3,H7	0.00	0.86
VxC-O4,H7	0.01	0.97	VxC-H8,H11	0.00	0.62	VCl-H2,H11	0.00	0.77
VxC-O4,H11	0.01	0.70	VxC-H7,H13	0.00	0.95	VCl-H3,H7	0.00	0.78
VCl-H8,H11	0.01	0.97	VxC-H9,H11	0.00	0.95	VxC-H3,H11	0.00	0.64
VCl-H9,H13	0.01	0.98	VCl-H7,H11	0.00	0.03	VxC-H2,H7	0.00	0.63
VCl-H7,H12	0.01	0.97	VCl-H11,H13	0.00	-0.05	VxC-C1,H8	0.00	0.10
VxC-C1,H12	0.01	0.25	VCl-H7,H9	0.00	-0.06	VxC-H2,H12	0.00	0.14
VxC-C1,H8	0.01	0.24	VxC-H9,H13	0.00	-0.94	VxC-H3,H8	0.00	-0.20
VCl-H9,C10	0.01	0.17	VxC-H7,H11	0.00	-0.99	VxC-H11,H12	0.00	-0.16
VCl-C6,H13	0.01	0.12	VxC-O5,H13	0.00	-0.36	VxC-H7,H8	0.00	-0.18
VxC-H3,H8	0.01	0.78	VxC-H11,H12	0.00	-0.09	VCl-H2,H13	0.00	-0.17
VxC-H2,H12	0.01	0.78	VxC-O4,H9	0.00	-0.38	VCl-H3,H9	0.00	-0.17
VxC-H3,H7	0.00	0.30	VxC-H7,H8	0.00	-0.10	VCl-H11,H13	0.00	-0.09
VxC-H2,H11	0.00	0.30	VCl-H9,H13	0.00	-0.91	VCl-H7,H9	0.00	-0.09
VxC-H9,H12	0.00	0.98	VCl-H8,H13	0.00	-0.61	VxC-H3,H9	0.00	-0.60
VxC-H8,H13	0.00	0.98	VCl-H9,H12	0.00	-0.61	VxC-H2,H13	0.00	-0.60
VxC-H7,C10	0.00	0.93	VxC-H7,C10	0.00	-0.95	VxC-H12,H13	0.00	-0.22
VxC-C6,H11	0.00	0.93	VxC-C6,H11	0.00	-0.95	VxC-H8,H9	0.00	-0.23
VxC-H7,H11	0.00	0.91	VCl-H3,H12	0.00	-0.55	VxC-H3,H12	0.00	-0.78
VCl-H9,H11	0.00	-0.01	VCl-H2,H8	0.00	-0.56	VxC-C1,H11	0.00	-0.23
VCl-H7,H13	0.00	-0.05	VxC-C1,H7	0.00	-0.99	VxC-H7,H11	0.00	-0.98
VxC-H7,H13	0.00	-0.98	VxC-C1,H11	0.00	-0.99	VxC-C1,H7	0.00	-0.24
VxC-H9,H11	0.00	-0.98	VCl-H7,H13	0.00	-0.97	VxC-O5,H9	0.00	-0.06
VxC-H8,H9	0.00	-0.42	VCl-H9,H11	0.00	-0.97	VxC-O4,H13	0.00	-0.07
VxC-H8,H11	0.00	-0.99	VxC-H2,H12	0.00	-0.96	VxC-H2,H8	0.00	-0.68
VxC-H7,H12	0.00	-0.99	VxC-H3,H8	0.00	-0.96	VCl-H7,H13	0.00	-0.69
VxC-H8,H12	0.00	-0.94	VxC-H2,H13	0.00	-0.63	VCl-H9,H11	0.00	-0.69

VxC-H12,H13	0.00	-0.72	VxC-H3,H9	0.00	-0.63	VCl-H7,H12	0.00	-0.94
VxC-H9,C10	0.00	-0.64	VCl-H8,H11	-0.01	-1.00	VCl-H2,H12	0.00	-0.93
VxC-C6,H13	0.00	-0.64	VCl-H2,H11	-0.01	-0.85	VxC-H9,H11	0.00	-0.94
VxC-H9,H13	0.00	-0.99	VCl-H3,H7	-0.01	-0.85	VxC-H7,H13	0.00	-0.94
VCl-H8,H12	0.00	-0.81	VCl-H7,H12	-0.01	-1.00	VCl-H8,H12	0.00	-0.71
VCl-H8,H9	0.00	-0.55	VCl-H8,H12	-0.01	-0.97	VCl-C10,H13	0.00	-0.04
VxC-H8,C10	0.00	-0.89	VxC-H2,H7	-0.01	-0.96	VCl-H8,H11	0.00	-0.91
VxC-C6,H12	0.00	-0.89	VxC-H3,H11	-0.01	-0.96	VCl-C6,H9	0.00	-0.04
VxC-O5,H9	0.00	-0.12	VxC-O5,H7	-0.01	-0.85	VCl-H3,H12	0.00	-0.94
VCl-H12,H13	0.00	-0.61	VCl-H3,H11	-0.01	-0.93	VCl-H3,H8	0.00	-0.90
VxC-O4,H13	-0.01	-0.24	VCl-H2,H7	-0.01	-0.93	VCl-H9,H13	0.00	-0.86
VCl-H9,H12	-0.01	-0.99	VxC-O4,H11	-0.01	-0.87	VxC-H7,H12	0.00	-0.75
VCl-H8,H13	-0.01	-0.99	VCl-H2,H9	-0.01	-0.95	VxC-H8,H11	0.00	-0.75
VxC-C1,H7	-0.01	-1.00	VCl-H3,H13	-0.01	-0.95	VxC-H9,H13	0.00	-0.89
VxC-O4,H9	-0.01	-0.81	Eintra-H9	-0.01	-0.34	VxC-O4,H9	0.00	-0.06
VxC-O5,H13	-0.01	-0.81	Eintra-H13	-0.01	-0.35	VxC-O5,H13	0.00	-0.06
VxC-C1,H11	-0.01	-1.00	VxC-H7,H9	-0.01	-0.98	VCl-C1,H11	0.00	-0.22
VCl-H7,H11	-0.01	-0.99	VxC-H11,H13	-0.01	-0.98	VCl-H2,H8	0.00	-0.91
VxC-O4,H8	-0.01	-0.91	VCl-H2,H12	-0.01	-0.99	VxC-C10,H13	0.00	-0.32
VxC-O5,H12	-0.01	-0.91	VCl-H3,H8	-0.01	-0.99	VCl-C1,H7	0.00	-0.25
VCl-O5,H13	-0.04	-0.37	VCl-H7,C10	-0.01	-0.76	VxC-C6,H9	0.00	-0.33
VxC-H2,O4	-0.04	-0.91	VxC-O5,H9	-0.01	-0.89	VCl-O5,H11	0.00	-0.29
VCl-O4,H9	-0.04	-0.41	VCl-C6,H11	-0.01	-0.76	VCl-O4,H7	0.00	-0.30
VxC-H3,O5	-0.04	-0.90	VxC-O4,H13	-0.01	-0.88	VCl-H3,H13	0.00	-0.97
VCl-H3,H12	-0.04	-0.98	VCl-H11,H12	-0.01	-1.00	VCl-H2,H9	0.00	-0.97
VCl-H2,H8	-0.04	-0.98	VCl-H7,H8	-0.01	-1.00	VxC-C6,H11	-0.01	-0.98
VCl-H7,H9	-0.06	-0.98	VxC-H8,H9	-0.02	-1.00	VxC-H7,C10	-0.01	-0.98
VCl-H11,H13	-0.06	-0.98	VxC-H12,H13	-0.02	-1.00	VCl-H11,H12	-0.01	-0.83
VxC-H2,H7	-0.06	-0.98	VCl-H12,H13	-0.02	-0.99	VCl-H7,H8	-0.01	-0.85
VxC-H3,H11	-0.06	-0.98	VCl-H8,H9	-0.02	-0.99	Eintra-H12	-0.01	-0.12
VxC-H7,H9	-0.09	-0.96	VxC-C1,C6	-0.03	-0.88	VxC-H3,O5	-0.01	-0.42
VxC-H11,H13	-0.09	-0.96	VxC-C1,C10	-0.03	-0.87	VxC-H2,O4	-0.01	-0.43
VCl-H7,C10	-0.09	-0.99	VCl-H2,H3	-0.03	-0.93	VCl-H12,H13	-0.01	-0.96
VCl-C6,H11	-0.09	-1.00	VxC-O5,H8	-0.04	-0.94	VCl-H8,H9	-0.01	-0.96
Eintra-H9	-0.09	-0.49	VxC-O4,H12	-0.04	-0.95	VxC-H2,H3	-0.01	-0.96
Eintra-H13	-0.09	-0.51	VCl-C6,H13	-0.04	-0.98	VCl-O5,C10	-0.02	-0.33
VCl-H2,H13	-0.10	-0.99	VxC-H2,H3	-0.04	-0.94	Eintra-H8	-0.02	-0.28
VCl-H3,H9	-0.10	-0.99	VCl-H9,C10	-0.04	-0.98	VCl-O4,C6	-0.02	-0.36
VCl-O5,H12	-0.12	-0.94	VCl-C6,H9	-0.04	-0.95	VxC-H2,O5	-0.02	-0.93
VCl-O4,H8	-0.13	-0.93	VCl-C10,H13	-0.04	-0.96	VxC-O4,H12	-0.02	-0.90
Eintra-H7	-0.14	-0.98	VxC-C1,H8	-0.06	-0.96	VxC-H3,O4	-0.02	-0.94
Eintra-H11	-0.15	-0.98	VxC-C1,H12	-0.06	-0.96	VxC-O5,H8	-0.02	-0.91
VxC-C10,H12	-0.15	-0.48	VCl-C1,H13	-0.06	-0.96	VxC-O4,H11	-0.03	-0.99
VxC-C6,H8	-0.15	-0.49	VCl-C1,H9	-0.06	-0.96	VxC-O5,H7	-0.03	-0.99
VxC-H2,H8	-0.23	-0.49	VxC-O5,C6	-0.07	-0.86	VCl-C6,H12	-0.03	-0.89
VxC-H3,H12	-0.23	-0.49	VxC-O4,C10	-0.07	-0.88	VCl-C6,H13	-0.03	-0.69
VCl-C6,H9	-0.26	-0.83	VCl-C1,C10	-0.08	-0.54	VCl-H9,C10	-0.03	-0.70
VCl-C10,H13	-0.27	-0.83	VCl-C1,C6	-0.09	-0.55	VCl-H2,O5	-0.03	-0.92

VxC-H3,H13	-0.28	-0.95	VCl-H8,C10	-0.09	-1.00	VCl-H3,O4	-0.03	-0.92
VxC-H2,H9	-0.28	-0.95	VCl-C6,H12	-0.09	-1.00	VCl-H3,O5	-0.04	-0.95
VCl-C1,H9	-0.29	-0.91	VCl-H2,C10	-0.09	-0.88	VCl-H8,C10	-0.04	-0.86
VCl-C1,H13	-0.30	-0.91	VCl-H3,C6	-0.09	-0.88	VCl-H2,O4	-0.04	-0.95
VxC-O4,O5	-0.34	-0.81	VCl-H3,C10	-0.12	-0.90	VCl-C1,H13	-0.04	-0.43
VCl-C10,H11	-0.40	-0.98	VCl-H2,C6	-0.12	-0.90	VCl-C1,H9	-0.04	-0.44
VCl-C6,H7	-0.41	-0.98	VCl-O5,H7	-0.12	-0.99	VCl-O5,H7	-0.04	-0.76
VCl-C1,H7	-0.50	-0.99	VCl-O4,H11	-0.12	-0.99	VCl-O4,H11	-0.04	-0.75
VCl-C1,H11	-0.50	-0.99	VxC-C10,H13	-0.13	-1.00	VxC-C1,H2	-0.05	-1.00
VCl-O4,H12	-0.69	-0.97	VxC-C6,H9	-0.13	-1.00	VxC-C1,H3	-0.05	-1.00
VCl-O4,O5	-0.70	-0.98	VxC-H2,C6	-0.15	-0.96	VxC-H9,C10	-0.05	-0.96
VCl-O5,H8	-0.70	-0.97	VxC-H3,C10	-0.15	-0.96	VxC-C6,H13	-0.05	-0.96
VxC-C10,H13	-0.88	-0.99	Eintra-H12	-0.16	-0.92	VxC-C6,H12	-0.06	-0.72
VxC-C6,H9	-0.88	-0.99	Eintra-H8	-0.16	-0.92	VxC-H8,C10	-0.06	-0.72
VCl-O4,C6	-0.91	-0.90	VxC-H2,H8	-0.17	-0.92	VCl-C10,H12	-0.08	-0.91
VCl-O5,C10	-0.95	-0.92	VxC-H3,H12	-0.17	-0.92	VCl-C6,H8	-0.09	-0.92
VxC-H3,C10	-1.20	-0.98	VCl-C10,H12	-0.22	-1.00	VxC-C6,C10	-0.11	-0.97
VxC-H2,C6	-1.20	-0.98	VCl-C6,H8	-0.22	-1.00	VCl-C1,H12	-0.11	-0.96
VxC-C1,H3	-1.79	-0.98	Eintra-O4	-0.30	-0.88	VxC-H9,H12	-0.12	-0.97
VxC-C1,H2	-1.80	-0.98	Eintra-O5	-0.30	-0.89	VxC-H8,H13	-0.12	-0.97
VCl-H2,O4	-1.93	-0.96	VCl-C1,H8	-0.31	-1.00	VCl-C1,H8	-0.13	-0.94
VCl-H3,O5	-1.93	-0.96	VCl-C1,H12	-0.31	-1.00	VCl-C1,C10	-0.19	-0.95
VCl-H3,O4	-1.97	-0.96	VxC-O4,O5	-0.34	-0.98	VCl-C1,C6	-0.20	-0.96
VCl-H2,O5	-1.97	-0.96	VCl-C6,C10	-0.39	-0.97	Eintra-O4	-0.30	-0.91
VCl-C1,C6	-3.79	-0.99	VCl-C1,H2	-0.45	-0.92	Eintra-O5	-0.33	-0.93
VCl-C1,C10	-3.81	-0.99	VCl-C1,H3	-0.45	-0.92	VCl-O4,O5	-0.36	-0.99
Eintra-O5	-4.48	-0.99	VCl-O4,O5	-0.55	-1.00	VCl-O5,C6	-0.56	-0.89
Eintra-O4	-4.70	-0.99	VCl-O5,C6	-0.66	-0.98	VCl-O4,C10	-0.57	-0.89
Eintra-C1	-13.52	-0.98	VCl-O4,C10	-0.67	-0.98	Eintra-C1	-0.57	-0.99

Table S2. REG values of all interactions in the FTHP-R isomer.

Segment 1			Segment 2		
**Contribution**	**REG**	**R**	**Contribution**	**REG**	**R**
VCl-C1,O15	1.33	0.82	Eintra-C5	0.62	0.99
Eintra-C5	1.29	0.99	Eintra-O15	0.57	0.97
Eintra-O15	1.18	0.97	VxC-C1,C2	0.49	0.99
VxC-C1,C2	1.14	1.00	VCl-C1,O15	0.37	0.76
VxC-C2,O15	0.48	0.99	VCl-C1,F16	0.33	0.97
VCl-C5,F16	0.46	0.61	VCl-H10,O15	0.26	0.91
Eintra-C3	0.41	0.96	VxC-C2,C3	0.20	0.94
VCl-C1,H10	0.37	0.91	VCl-C5,F16	0.20	0.65
VCl-O15,F16	0.37	0.99	VxC-C2,O15	0.17	0.95
VxC-C5,F16	0.36	0.92	VCl-C2,O15	0.17	1.00
VCl-C2,O15	0.35	0.99	Eintra-H8	0.17	1.00
Eintra-H14	0.35	0.93	Eintra-H14	0.17	0.99
VCl-C5,H10	0.32	0.87	Eintra-H7	0.17	1.00
Eintra-H7	0.32	1.00	VxC-H9,F16	0.14	0.94
VxC-H10,H11	0.29	0.96	VCl-C5,H14	0.14	0.96
VCl-C4,C5	0.28	0.99	VxC-C5,F16	0.13	0.99
Eintra-H9	0.28	1.00	VCl-C1,H9	0.13	0.85
VxC-C1,C4	0.27	0.95	Eintra-H10	0.12	0.95
VxC-C3,H14	0.27	0.98	VxC-C1,F16	0.11	0.97
VCl-H8,O15	0.26	0.84	Eintra-H9	0.11	0.95
Eintra-H10	0.26	0.98	VCl-H10,F16	0.11	0.95
VCl-C1,F16	0.25	1.00	VCl-C2,F16	0.11	1.00
VCl-C3,C5	0.25	0.96	VxC-C1,C3	0.10	0.98
VCl-C3,C4	0.23	0.99	VCl-C3,C5	0.10	0.99
VCl-C5,H14	0.22	0.93	VCl-C4,C5	0.09	0.88
VxC-C4,H10	0.21	0.98	VCl-O15,F16	0.09	0.72
VxC-C1,C3	0.20	0.97	VCl-C5,H7	0.06	0.78
VCl-C5,H13	0.17	0.68	Eintra-H6	0.06	1.00
VCl-H7,O15	0.17	0.98	VxC-H7,H11	0.06	0.98
VxC-H8,F16	0.16	0.84	VCl-C5,H9	0.06	0.54
VxC-H8,O15	0.16	0.93	VxC-H8,O15	0.05	0.99
VxC-C1,O15	0.15	0.69	VxC-C4,H7	0.05	0.99
VxC-C2,F16	0.15	0.86	VxC-C1,O15	0.05	0.81
VCl-C2,C3	0.14	0.91	VCl-C1,H14	0.05	0.96
VxC-C1,H11	0.12	0.87	Eintra-C3	0.05	0.86
VCl-C5,H6	0.11	0.96	VCl-C5,H12	0.05	0.92
VxC-C4,C5	0.10	0.99	VxC-C1,H14	0.05	0.98
VCl-C2,F16	0.09	0.99	VCl-C3,C4	0.04	0.99
VCl-H9,O15	0.09	0.55	VxC-H7,H9	0.04	0.97
VxC-H7,O15	0.09	0.96	VCl-H11,O15	0.04	0.97
VCl-H7,F16	0.08	0.98	VCl-H13,F16	0.04	0.95
VxC-C3,H10	0.07	0.88	VxC-C3,H10	0.04	0.99
VxC-H6,H11	0.07	0.95	VxC-C1,H8	0.04	0.90
VxC-C2,C5	0.07	0.85	VxC-C5,H13	0.03	0.98

VxC-C5,H13	0.07	0.99	VxC-C4,F16	0.03	0.99
VxC-H8,H10	0.07	0.96	VxC-H9,H14	0.03	0.92
VxC-C3,H9	0.06	0.98	VxC-C2,C4	0.03	0.79
VxC-H6,H8	0.06	0.94	VCl-C3,H14	0.03	0.95
VxC-C1,H14	0.06	0.96	Eintra-H13	0.03	0.88
VCl-C1,H14	0.06	0.77	Eintra-H12	0.02	0.86
VCl-H8,F16	0.06	0.64	VCl-H11,F16	0.02	0.95
VCl-C1,H6	0.05	0.91	VxC-C5,H10	0.02	0.98
VCl-C1,C3	0.05	0.97	VxC-H7,O15	0.02	0.52
VxC-H12,H14	0.05	0.95	VxC-C3,H9	0.02	0.69
VCl-C3,H6	0.05	0.86	VCl-C2,H7	0.02	0.68
VxC-C4,H6	0.05	0.99	VxC-H12,F16	0.02	0.97
VxC-C4,F16	0.05	0.98	VCl-C1,C3	0.02	0.97
VxC-C4,H7	0.04	0.86	VCl-H13,O15	0.02	0.42
VCl-H9,F16	0.04	0.72	VxC-C2,H9	0.02	0.93
VxC-C1,C5	0.04	0.99	VxC-C2,H11	0.02	0.64
VCl-H12,F16	0.04	0.81	VCl-C5,H6	0.02	0.96
VCl-C4,H14	0.04	0.97	VxC-H6,H8	0.02	1.00
VxC-C5,H11	0.03	0.93	VxC-C1,C5	0.01	0.94
Eintra-H13	0.03	0.66	VxC-H7,H14	0.01	0.92
VxC-H13,F16	0.03	0.72	VxC-C1,H6	0.01	0.93
VCl-H12,O15	0.03	0.71	VCl-C4,H14	0.01	0.96
VxC-C1,H8	0.03	0.90	VCl-C1,H12	0.01	0.77
VxC-C3,H6	0.03	0.40	VxC-C4,H8	0.01	0.89
VxC-H7,H9	0.03	0.84	VCl-C3,H8	0.01	0.90
VxC-H6,H14	0.03	1.00	VCl-C1,H7	0.01	0.26
VCl-H9,H10	0.03	0.94	VCl-C3,H12	0.01	0.99
VCl-C4,H11	0.03	0.96	VxC-C4,H12	0.01	0.48
VxC-C5,H9	0.02	0.90	Eintra-C4	0.01	0.22
VxC-C4,H12	0.02	0.36	VxC-C5,O15	0.01	0.16
VCl-H7,H10	0.02	0.98	VCl-C3,H7	0.01	0.97
VCl-C5,H11	0.02	0.33	VCl-H7,H10	0.01	0.95
VCl-C3,H7	0.02	0.95	VCl-H8,F16	0.01	0.34
VCl-C4,H6	0.02	0.99	VCl-C4,H12	0.01	0.98
VCl-C4,H10	0.02	0.83	VxC-H11,H13	0.01	0.90
VCl-H10,H13	0.02	0.88	VCl-C3,H9	0.01	0.83
VxC-H11,H12	0.02	0.98	VxC-H6,H10	0.01	1.00
VCl-C3,H13	0.01	0.91	VCl-H13,H14	0.01	0.93
VCl-C3,H10	0.01	0.75	VCl-H8,H14	0.01	1.00
VCl-C4,H13	0.01	0.85	VCl-H6,H14	0.01	0.97
VCl-H6,H14	0.01	0.90	VCl-C3,H6	0.01	0.90
VCl-C1,C4	0.01	0.49	VCl-H6,H7	0.01	1.00
VCl-C2,C4	0.01	0.98	VxC-C5,H11	0.01	0.60
VxC-C4,H9	0.01	0.95	VCl-H12,H14	0.01	0.99
VCl-C3,H12	0.01	0.93	VCl-H8,H9	0.01	0.95
VxC-H9,H14	0.01	0.98	VCl-H7,H13	0.00	0.81
VCl-H13,H14	0.01	0.96	VCl-C5,H8	0.00	0.13
VCl-H10,H14	0.01	0.96	VxC-C4,H13	0.00	0.64

VxC-H10,H12	0.01	0.98	VCl-H7,H9	0.00	0.59
VCl-H10,H12	0.01	0.93	VCl-H7,H8	0.00	0.81
VxC-C1,H12	0.01	0.94	VxC-H6,H11	0.00	0.56
VCl-H7,H11	0.01	0.98	VxC-H10,H13	0.00	1.00
VCl-C3,H11	0.01	0.98	VxC-H11,O15	0.00	0.14
VCl-H6,H12	0.01	0.93	VCl-H9,H13	0.00	0.57
VxC-C4,H13	0.01	0.84	VCl-C3,H13	0.00	0.78
VCl-C3,H8	0.01	0.50	VCl-H7,H14	0.00	0.75
VxC-H6,H10	0.01	0.70	VxC-C1,H13	0.00	0.47
VxC-C5,H8	0.01	0.83	VCl-H7,H12	0.00	0.79
VxC-H7,H12	0.01	0.98	VCl-C4,H9	0.00	0.54
VCl-H6,H8	0.01	0.98	VCl-H9,H12	0.00	0.72
VCl-C3,H9	0.01	0.90	VCl-H12,H13	0.00	0.94
VxC-H11,F16	0.01	0.96	VCl-H10,H11	0.00	0.67
VxC-H7,F16	0.01	0.80	VxC-H7,F16	0.00	0.97
VxC-H11,H13	0.01	0.95	VxC-H8,H12	0.00	0.90
VxC-H9,H11	0.01	0.84	VxC-C5,H7	0.00	1.00
VxC-C5,H12	0.01	0.74	VCl-C4,H6	0.00	0.88
VCl-H6,H13	0.01	0.97	VxC-H7,H12	0.00	1.00
VCl-H11,H14	0.01	0.99	VCl-H11,H14	0.00	0.66
VCl-H6,H9	0.00	0.91	VxC-H12,O15	0.00	0.78
VxC-H8,H14	0.00	0.15	VCl-H6,H12	0.00	0.97
VCl-C1,H11	0.00	0.21	VxC-H10,H14	0.00	0.89
VCl-H9,H14	0.00	0.72	VxC-H8,H13	0.00	0.96
VxC-C5,H7	0.00	0.91	VxC-H11,F16	0.00	0.96
VCl-C2,H11	0.00	0.87	VCl-C4,H7	0.00	0.25
VCl-C4,H9	0.00	0.69	VxC-H6,H14	0.00	0.19
VxC-C4,H14	0.00	0.20	VxC-C5,H8	0.00	0.99
VCl-H12,H14	0.00	0.55	VCl-H8,O15	0.00	0.02
VxC-H7,H13	0.00	0.98	VCl-H8,H12	0.00	0.81
VCl-H8,H10	0.00	0.24	VxC-C3,H11	0.00	0.12
VxC-C2,H13	0.00	0.98	VCl-C4,H8	0.00	0.25
VxC-C3,H11	0.00	0.25	VCl-H6,H13	0.00	0.82
VCl-C2,H10	0.00	0.06	VxC-H10,H12	0.00	0.84
VCl-H11,H13	0.00	0.21	VCl-H6,H9	0.00	0.65
VxC-H10,H14	0.00	0.43	VCl-H10,H14	0.00	0.09
VxC-H12,H13	0.00	0.24	VCl-C2,H8	0.00	0.04
VCl-H6,H7	0.00	0.38	VxC-H7,H13	0.00	0.55
VxC-H9,H12	0.00	0.95	VxC-H9,H11	0.00	0.14
VxC-H10,H13	0.00	0.35	VxC-C2,C5	0.00	0.20
Eintra-C4	0.00	0.00	VxC-C3,H13	0.00	0.08
VxC-C3,H13	0.00	0.14	VxC-H6,H13	0.00	0.00
VCl-H9,H11	0.00	0.00	VxC-H8,H11	0.00	-0.01
VxC-C2,H14	0.00	-0.02	VxC-C2,H13	0.00	-0.49
VxC-H8,H13	0.00	-0.26	VxC-C2,H14	0.00	-0.03
VxC-C5,H10	0.00	-0.05	VCl-C1,H6	0.00	-0.40
VCl-C2,H6	0.00	-0.21	VCl-H8,H13	0.00	-0.13
VxC-H6,H13	0.00	-0.71	VxC-H9,H12	0.00	-0.99

VxC-H13,H14	0.00	-0.97	VCl-H6,H10	0.00	-0.84
VxC-H9,F16	0.00	-0.53	VCl-C3,H11	0.00	-0.75
VCl-H12,H13	0.00	-0.97	VxC-C4,H9	0.00	-0.46
VxC-C1,F16	0.00	-0.45	VxC-H13,H14	0.00	-0.98
VxC-H9,H13	0.00	-0.93	VxC-H8,F16	0.00	-0.59
VCl-H9,H13	0.00	-0.73	VxC-H14,O15	0.00	-0.78
VCl-H11,F16	0.00	-0.13	VxC-H11,H12	0.00	-0.36
VxC-H12,O15	0.00	-0.55	VxC-C2,H12	0.00	-0.83
VxC-H6,O15	0.00	-0.97	VxC-H6,O15	0.00	-0.89
VxC-H10,F16	0.00	-0.92	VxC-C5,H6	0.00	-0.96
VCl-H8,H11	0.00	-0.82	VCl-H8,H11	0.00	-0.95
VxC-H8,H11	0.00	-0.94	VCl-H6,H11	0.00	-0.94
VCl-H9,H12	0.00	-0.80	VCl-H11,H12	0.00	-0.95
VCl-C4,H7	0.00	-0.96	VxC-H11,H14	0.00	-0.97
VCl-H11,H12	0.00	-0.96	VCl-H9,H14	0.00	-0.75
VxC-H6,H9	0.00	-0.91	VxC-C4,O15	0.00	-0.07
VCl-H7,H12	-0.01	-0.98	VCl-C4,H13	0.00	-0.42
VCl-C2,H14	-0.01	-0.93	VCl-H9,H11	0.00	-0.99
VxC-H6,F16	-0.01	-0.94	VxC-H6,H9	0.00	-0.90
VxC-H6,H7	-0.01	-0.42	VxC-H6,H12	0.00	-0.66
VCl-C2,H8	-0.01	-0.78	VxC-H12,H14	0.00	-0.47
VCl-H8,H14	-0.01	-0.99	VxC-C4,H6	0.00	-0.45
VCl-H11,O15	-0.01	-0.19	VCl-C2,H11	0.00	-0.96
VCl-H7,H14	-0.01	-0.98	VCl-C2,H14	0.00	-0.91
VCl-H7,H13	-0.01	-0.98	VCl-H10,H12	0.00	-0.86
VxC-C2,H10	-0.01	-0.33	VCl-H6,H8	0.00	-0.92
VxC-H14,O15	-0.01	-0.99	VxC-C1,H12	0.00	-0.96
VCl-H6,H10	-0.01	-0.88	VxC-H6,F16	0.00	-0.95
VCl-H8,H12	-0.01	-0.92	VxC-H9,H13	0.00	-1.00
VCl-H8,H9	-0.01	-0.80	VxC-H10,F16	0.00	-0.90
VCl-H6,H11	-0.01	-0.93	VCl-C2,H12	0.00	-0.96
VCl-H8,H13	-0.01	-0.80	VCl-C2,H9	0.00	-0.48
VxC-C2,H9	-0.01	-0.70	VxC-C5,H12	0.00	-0.88
VCl-C4,H8	-0.01	-0.82	VCl-H11,H13	0.00	-0.97
VxC-C3,H12	-0.01	-0.98	VxC-H12,H13	0.00	-0.97
VxC-H8,H12	-0.01	-0.99	VxC-C4,H10	0.00	-0.75
VCl-C2,H13	-0.01	-0.97	VCl-C4,H11	-0.01	-0.78
VCl-C2,H12	-0.01	-1.00	VxC-C3,H12	-0.01	-0.99
VxC-H12,F16	-0.01	-0.64	VxC-C5,H14	-0.01	-0.84
VxC-C5,H6	-0.02	-0.88	VCl-H7,H11	-0.01	-0.94
VCl-H13,F16	-0.02	-0.29	VCl-C3,H10	-0.01	-0.75
VxC-C1,H7	-0.02	-0.32	VxC-C2,F16	-0.01	-0.93
VCl-H7,H9	-0.02	-0.99	VCl-C1,H8	-0.01	-0.51
VCl-C4,H12	-0.02	-0.99	VxC-H8,H10	-0.01	-0.45
VxC-H11,O15	-0.02	-0.40	VCl-H8,H10	-0.01	-0.92
VCl-H10,H11	-0.02	-0.92	VCl-C2,H6	-0.01	-1.00
VxC-H7,H11	-0.02	-0.90	VxC-H10,H11	-0.01	-0.74
VCl-C1,H13	-0.02	-0.73	VxC-C4,H14	-0.01	-0.73

VxC-C2,H12	-0.02	-0.95	VCl-H6,F16	-0.01	-0.96
VxC-H11,H14	-0.02	-0.90	VCl-C2,H13	-0.01	-1.00
VCl-H7,H8	-0.02	-0.96	VCl-H9,H10	-0.01	-0.97
VxC-C1,H13	-0.02	-0.78	VCl-H10,H13	-0.01	-0.91
VxC-H9,H10	-0.02	-0.99	VxC-C5,H9	-0.01	-0.85
Eintra-H6	-0.03	-0.96	VCl-C4,F16	-0.01	-0.59
VxC-H6,H12	-0.03	-0.97	VCl-H6,O15	-0.01	-0.96
VCl-C2,H9	-0.03	-0.98	VxC-C2,H6	-0.01	-0.68
VxC-C2,H6	-0.03	-0.81	VCl-C4,H10	-0.01	-0.92
VCl-C5,H12	-0.04	-0.65	VxC-C3,H7	-0.01	-0.62
VxC-C4,H11	-0.04	-0.95	VxC-C3,H6	-0.01	-0.57
Eintra-H8	-0.04	-0.43	VxC-C2,H10	-0.02	-0.98
VCl-H6,F16	-0.04	-0.97	VCl-C2,C3	-0.02	-0.65
VCl-C1,H12	-0.05	-0.88	VCl-C2,C4	-0.02	-0.99
VxC-C5,H14	-0.05	-0.98	VxC-H7,H8	-0.02	-1.00
VCl-C5,H9	-0.05	-0.53	VxC-C4,H11	-0.02	-0.97
VCl-C3,H14	-0.05	-0.98	VCl-C1,C4	-0.02	-0.97
VxC-H7,H8	-0.05	-0.98	VxC-C3,O15	-0.02	-0.99
Eintra-H12	-0.06	-0.99	VxC-H9,H10	-0.02	-0.99
VxC-H9,O15	-0.06	-1.00	VCl-H7,F16	-0.02	-0.65
VxC-C3,H8	-0.06	-0.99	VxC-C3,H8	-0.02	-0.98
VxC-C3,H7	-0.06	-0.95	VCl-H12,F16	-0.02	-0.88
VxC-C1,H6	-0.07	-1.00	VxC-C3,C5	-0.02	-0.85
VxC-H13,O15	-0.07	-0.93	VCl-C2,H10	-0.02	-0.95
VxC-H7,H14	-0.08	-1.00	VCl-C4,O15	-0.03	-0.66
VxC-C2,H7	-0.08	-0.69	VxC-H13,O15	-0.03	-0.90
VxC-C4,O15	-0.08	-0.72	VxC-C1,H11	-0.03	-0.89
VxC-C4,H8	-0.08	-1.00	VCl-C1,H13	-0.03	-1.00
VxC-C1,H9	-0.08	-0.75	VCl-C1,H11	-0.03	-0.99
VxC-C3,O15	-0.09	-0.96	VxC-C1,H10	-0.03	-0.86
VxC-C2,H11	-0.09	-0.99	Eintra-H11	-0.03	-0.99
VxC-H10,O15	-0.10	-0.97	VxC-C1,H7	-0.03	-0.92
VCl-C1,H7	-0.10	-0.98	VxC-H13,F16	-0.04	-0.99
VCl-C2,H7	-0.11	-0.99	VCl-H12,O15	-0.04	-0.92
VxC-C3,C5	-0.11	-0.98	VCl-C5,H11	-0.04	-0.92
VCl-H14,F16	-0.11	-0.93	VxC-H8,H14	-0.04	-0.96
VCl-H6,O15	-0.12	-0.94	VxC-H9,O15	-0.04	-0.95
VxC-H8,H9	-0.12	-1.00	VxC-C3,H14	-0.04	-0.98
VCl-C4,F16	-0.13	-0.99	VxC-H6,H7	-0.05	-1.00
VxC-C2,C4	-0.14	-0.99	VCl-C3,F16	-0.05	-0.99
VCl-C5,H7	-0.15	-0.98	VCl-C5,H13	-0.05	-0.74
VxC-C3,F16	-0.15	-0.98	VxC-H10,O15	-0.05	-1.00
VCl-C3,F16	-0.15	-0.98	VxC-H8,H9	-0.05	-0.97
VCl-H13,O15	-0.15	-0.73	VxC-C4,C5	-0.06	-0.96
VCl-H10,F16	-0.15	-0.87	VCl-H9,O15	-0.06	-0.47
VCl-C1,H9	-0.15	-0.74	VCl-H9,F16	-0.06	-0.81
VxC-O15,F16	-0.17	-0.82	VxC-H7,H10	-0.07	-1.00
VxC-H7,H10	-0.18	-1.00	VxC-C3,F16	-0.07	-0.90

VCl-H14,O15	-0.18	-0.91	VxC-C1,C4	-0.07	-0.94
VxC-C5,O15	-0.19	-0.63	VCl-C3,O15	-0.07	-0.98
VCl-C5,H8	-0.20	-0.81	VxC-C3,C4	-0.08	-0.99
VCl-C4,O15	-0.21	-0.99	VCl-H14,F16	-0.09	-0.96
VCl-C3,O15	-0.21	-0.95	VCl-H7,O15	-0.10	-0.85
VCl-C2,C5	-0.23	-0.99	VxC-H14,F16	-0.10	-0.87
VCl-C1,H8	-0.24	-0.94	VCl-H14,O15	-0.11	-0.96
Eintra-F16	-0.26	-0.58	Eintra-C1	-0.12	-0.54
Eintra-H11	-0.30	-0.99	VxC-C2,H7	-0.13	-0.97
VxC-C2,H8	-0.32	-0.91	VxC-C2,H8	-0.14	-0.95
Eintra-C2	-0.34	-0.94	VxC-C1,H9	-0.15	-0.95
VxC-C2,C3	-0.37	-0.80	VxC-O15,F16	-0.17	-0.92
VxC-C1,H10	-0.39	-0.99	VCl-C2,C5	-0.17	-1.00
VCl-H10,O15	-0.39	-0.88	VCl-C5,H10	-0.19	-0.92
VCl-C1,C5	-0.44	-0.92	Eintra-F16	-0.20	-0.80
VxC-H14,F16	-0.48	-0.98	VCl-C1,C2	-0.23	-0.99
VCl-C1,C2	-0.60	-1.00	VCl-C1,H10	-0.25	-0.94
VxC-C3,C4	-0.71	-0.97	Eintra-C2	-0.27	-1.00
Eintra-C1	-1.00	-0.85	VCl-C1,C5	-0.31	-0.88
VCl-C5,O15	-3.69	-0.96	VCl-C5,O15	-1.47	-0.99

Table S3. REG values of all interactions in the FTHP-S isomer.

Segment 1			Segment 2		
**Contribution**	**REG**	**R**	**Contribution**	**REG**	**R**
Eintra-C1	1.56	0.95	VCl-C5,F16	3.73	0.99
Eintra-C5	1.45	0.99	Eintra-O15	1.06	0.93
VxC-C5,O15	0.96	0.95	VxC-C1,O15	0.78	0.98
VCl-C1,F16	0.94	0.94	VCl-C1,O15	0.70	0.75
VCl-C1,C2	0.82	1.00	VCl-C5,H13	0.66	0.97
VxC-C3,O15	0.76	1.00	VxC-C5,F16	0.62	0.99
VCl-H9,O15	0.73	0.97	VxC-C4,C5	0.37	0.99
VxC-C1,O15	0.66	0.97	Eintra-H13	0.35	0.97
Eintra-C2	0.56	0.99	VCl-C5,H9	0.30	0.91
VCl-C4,C5	0.54	0.99	VCl-C1,H9	0.29	0.89
VCl-H13,O15	0.48	0.94	VxC-C2,O15	0.29	0.99
VxC-H14,O15	0.40	1.00	Eintra-H11	0.28	1.00
VxC-C1,H9	0.39	0.96	Eintra-H9	0.22	0.93
VxC-H10,F16	0.36	0.86	VCl-O15,F16	0.20	0.98
VxC-C2,H8	0.36	1.00	VxC-C4,O15	0.20	0.97
VCl-H9,F16	0.35	0.99	VCl-H10,O15	0.14	0.84
Eintra-C4	0.33	0.98	VxC-H7,O15	0.13	0.98
VCl-C3,O15	0.33	0.99	VCl-C1,H13	0.12	0.98
VCl-H13,F16	0.30	0.98	Eintra-C3	0.12	0.99
VCl-C2,C5	0.29	1.00	VCl-C5,H11	0.12	1.00
VxC-C1,F16	0.28	0.85	VxC-C1,C5	0.11	0.96
VCl-H14,O15	0.27	0.99	VCl-C1,C2	0.11	0.94
VxC-C4,O15	0.23	1.00	Eintra-H8	0.11	0.96
Eintra-F16	0.22	0.26	VCl-H7,F16	0.11	0.99
VxC-C2,H7	0.22	1.00	VCl-C3,C5	0.10	0.98
VCl-C1,H10	0.19	0.74	VCl-H7,O15	0.10	0.99
VCl-C3,F16	0.18	0.99	VxC-H9,H13	0.10	0.94
VCl-H14,F16	0.16	1.00	VxC-C4,H11	0.10	0.98
VxC-C5,H13	0.15	0.91	VxC-H11,O15	0.09	0.98
VCl-C5,H12	0.15	1.00	VCl-C3,C4	0.09	0.99
VxC-C4,H11	0.14	0.98	Eintra-H12	0.08	1.00
VCl-C1,H8	0.13	0.98	VCl-C5,H14	0.07	0.91
Eintra-H11	0.12	0.94	VCl-C5,H8	0.07	0.98
VCl-C5,H8	0.12	0.95	VxC-C1,F16	0.07	0.95
VxC-C1,C5	0.12	0.96	VxC-C5,H9	0.07	0.90
VCl-O15,F16	0.11	0.45	VCl-C2,C3	0.06	0.99
VCl-C5,H11	0.09	0.98	Eintra-H10	0.06	0.94
VxC-H7,H10	0.09	0.99	VxC-C3,C5	0.06	0.98
VCl-C3,C4	0.07	0.99	VxC-C3,H9	0.06	0.90
VxC-C2,O15	0.07	0.64	VxC-H12,F16	0.06	1.00
VxC-C3,H14	0.07	0.97	VxC-H9,H14	0.05	0.75
VxC-C5,H9	0.07	0.99	VCl-H10,F16	0.05	0.89
VxC-C1,H10	0.06	0.63	VCl-C1,C3	0.05	0.98
VCl-C2,H10	0.06	1.00	VCl-C1,H11	0.05	1.00

VxC-C1,H13	0.06	0.92	VxC-H13,H14	0.04	0.99
VCl-C1,C4	0.06	0.99	Eintra-C2	0.04	0.72
VxC-C4,H12	0.06	0.93	VCl-C1,H8	0.04	1.00
VxC-H8,H9	0.05	0.97	VCl-C3,H14	0.04	0.92
VCl-C2,C3	0.05	0.83	VxC-H12,O15	0.04	0.99
VxC-C3,H8	0.05	0.92	VxC-C4,H13	0.04	0.93
VCl-C1,H7	0.05	0.92	VxC-C3,H13	0.03	0.98
Eintra-H13	0.05	0.31	VCl-C1,F16	0.03	0.74
VCl-C1,H12	0.05	0.99	VxC-C2,H8	0.03	0.91
Eintra-H6	0.05	0.99	VxC-C5,H11	0.03	0.98
VCl-C2,C4	0.05	0.99	VCl-C2,H9	0.03	0.94
VxC-C2,H6	0.04	0.99	VCl-C4,H13	0.03	0.96
VCl-C5,H6	0.04	0.95	VxC-C3,F16	0.03	0.97
VCl-H7,O15	0.04	0.84	VxC-H11,H13	0.03	0.98
VxC-C3,H7	0.04	0.99	VCl-H12,O15	0.03	0.97
VxC-H7,H8	0.04	0.99	VxC-C5,H10	0.03	0.89
VxC-H6,H7	0.04	0.95	Eintra-H7	0.02	0.69
VxC-C1,C4	0.04	0.86	VxC-C1,C3	0.02	0.44
VxC-H8,H14	0.03	0.89	VxC-C3,H8	0.02	1.00
VxC-H6,O15	0.03	1.00	VCl-C1,H14	0.02	0.75
VCl-C1,H6	0.03	0.99	VCl-C3,H11	0.02	1.00
VCl-C2,H8	0.02	0.73	VCl-C2,H8	0.02	0.96
VxC-H11,O15	0.02	0.98	VCl-H11,H13	0.02	0.98
VxC-C3,H12	0.02	0.98	VCl-C3,H13	0.02	0.97
VxC-C2,F16	0.02	1.00	VxC-H8,H14	0.02	0.99
Eintra-H12	0.02	0.92	VCl-H14,O15	0.02	0.63
VCl-C2,H6	0.02	1.00	VxC-C5,H14	0.02	0.99
VCl-C4,H12	0.02	0.96	VxC-C2,H14	0.02	0.97
VCl-H8,H10	0.02	1.00	VxC-C1,H13	0.02	0.37
VCl-C4,H6	0.02	0.98	VCl-C3,H8	0.02	0.97
VxC-C3,H6	0.02	0.86	VCl-H9,H10	0.01	0.87
VCl-H9,H14	0.01	0.92	VCl-C2,H13	0.01	0.99
VCl-C1,H11	0.01	0.99	VCl-C4,H9	0.01	0.91
VCl-C2,H12	0.01	1.00	VCl-C2,C4	0.01	1.00
VCl-H11,H12	0.01	1.00	VxC-H8,O15	0.01	0.90
VCl-C2,H11	0.01	1.00	VxC-C4,H14	0.01	0.98
VxC-H11,H12	0.01	0.99	VxC-H8,H10	0.01	0.90
VCl-C2,H9	0.01	0.58	VCl-C3,H9	0.01	0.85
VxC-H12,H14	0.01	0.93	VCl-C2,C5	0.01	0.63
VCl-C4,H8	0.01	0.97	VCl-C4,H12	0.01	0.79
VxC-H9,H13	0.01	0.98	VxC-H12,H14	0.01	0.99
VCl-H7,H8	0.01	0.99	VCl-H8,H9	0.01	0.95
VxC-C3,H13	0.01	0.96	VCl-H9,H11	0.01	0.93
VxC-H8,H10	0.01	0.46	VxC-H6,H14	0.01	0.99
VCl-C4,H10	0.01	0.77	VCl-C4,H14	0.01	0.96
VCl-C4,H7	0.01	0.99	VCl-H12,H13	0.01	1.00
VCl-H12,H13	0.01	0.94	VxC-C4,F16	0.01	0.22
VCl-H8,H11	0.00	0.95	VxC-C1,H10	0.01	0.12

VCl-H6,H11	0.00	0.98	VxC-H6,H7	0.01	0.87
VCl-H6,H8	0.00	0.92	VxC-H10,F16	0.01	0.98
VCl-H8,H13	0.00	0.83	VCl-C5,H6	0.01	0.57
VxC-H7,O15	0.00	0.12	VxC-C4,H9	0.01	1.00
VCl-H7,H11	0.00	0.97	VCl-C4,H11	0.01	0.44
VCl-C2,H13	0.00	0.62	VCl-H10,H13	0.01	0.99
VxC-C4,H10	0.00	0.84	VCl-C2,H10	0.01	0.82
VCl-H8,H12	0.00	0.99	VCl-H11,H12	0.01	1.00
VCl-H6,H12	0.00	0.99	VCl-C4,F16	0.01	0.53
VxC-C1,H12	0.00	0.94	VCl-H6,H9	0.00	0.93
VCl-H10,H12	0.00	0.75	VxC-C2,H13	0.00	1.00
VxC-C1,H11	0.00	0.74	VCl-H11,H14	0.00	0.93
VCl-H6,H10	0.00	0.92	VCl-C4,H8	0.00	0.97
VxC-C4,H14	0.00	0.18	VCl-C3,H12	0.00	0.99
VxC-H10,H11	0.00	0.88	VCl-H6,H13	0.00	0.98
VxC-H6,H13	0.00	0.97	VCl-C1,H6	0.00	0.98
VCl-C2,H7	0.00	0.06	VCl-H8,H13	0.00	0.96
VxC-H6,H9	0.00	0.54	VCl-H8,H11	0.00	0.99
VxC-C2,H13	0.00	0.98	VCl-H9,H12	0.00	0.92
VCl-H7,H13	0.00	0.45	VCl-C4,H6	0.00	0.97
VxC-H12,H13	0.00	0.12	VxC-C3,H10	0.00	0.62
VxC-C4,H8	0.00	0.49	VCl-C2,H11	0.00	0.98
VxC-H7,F16	0.00	0.06	VCl-H10,H14	0.00	0.86
VCl-H7,H12	0.00	0.35	VxC-H6,H9	0.00	0.94
VCl-H6,H13	0.00	0.17	VCl-H6,H14	0.00	0.86
VxC-H14,F16	0.00	0.17	VxC-H6,F16	0.00	1.00
VxC-H9,H12	0.00	0.94	VCl-H6,H8	0.00	1.00
VxC-C4,H9	0.00	0.03	VxC-H7,H13	0.00	1.00
VxC-H8,H12	0.00	-0.07	VxC-H6,H10	0.00	0.93
VCl-C4,H13	0.00	-0.02	VxC-H7,H9	0.00	0.50
VxC-C2,H12	0.00	-0.41	VxC-C1,C4	0.00	0.77
VxC-H8,H11	0.00	-0.51	VxC-H9,H11	0.00	0.99
VxC-H8,H13	0.00	-0.93	VxC-H8,F16	0.00	1.00
VxC-H7,H13	0.00	-0.91	VCl-C2,H14	0.00	0.63
VxC-C4,H6	0.00	-0.51	VCl-H6,H11	0.00	1.00
VCl-H8,H9	0.00	-0.17	VCl-H10,H11	0.00	0.87
VxC-H8,F16	0.00	-0.96	VCl-H7,H9	0.00	0.69
VCl-H10,H11	0.00	-0.38	VCl-H8,H12	0.00	0.99
VxC-C1,H7	0.00	-0.25	VxC-C3,H12	0.00	0.29
VCl-C3,H8	0.00	-0.23	VCl-C3,H6	0.00	0.25
VxC-H10,H12	0.00	-0.99	VCl-H8,H10	0.00	0.62
VxC-C5,H8	0.00	-0.82	VCl-C3,H10	0.00	0.37
VxC-C2,H14	0.00	-0.50	VxC-H6,H13	0.00	0.97
VxC-H6,H12	0.00	-0.92	VCl-H7,H14	0.00	0.96
VxC-H9,H11	0.00	-1.00	VxC-H9,H12	0.00	0.99
VxC-H7,H14	0.00	-0.60	VxC-C4,H10	0.00	0.98
VxC-H11,H14	0.00	-0.97	VCl-H6,H12	0.00	0.97
VxC-H6,F16	0.00	-0.99	VxC-H14,F16	0.00	0.61

VCl-H7,H14	0.00	-0.99	VxC-H10,O15	0.00	0.01
VCl-H9,H12	0.00	-0.91	VxC-H6,H11	0.00	0.20
VCl-H7,F16	0.00	-0.11	VxC-H10,H11	0.00	0.99
VCl-C3,H12	0.00	-0.86	VxC-H10,H12	0.00	0.64
VCl-H6,H7	0.00	-0.91	VxC-C1,H6	0.00	0.10
VCl-H6,H9	0.00	-0.87	VCl-H12,H14	0.00	0.08
VxC-H7,H12	0.00	-0.89	VxC-C1,H12	0.00	-0.37
VCl-C3,H7	-0.01	-0.95	VxC-H8,H13	0.00	-1.00
VxC-H6,H11	-0.01	-0.97	VxC-H10,H14	0.00	-0.31
VCl-H7,H9	-0.01	-0.98	VxC-C5,H8	0.00	-0.97
VCl-H7,H10	-0.01	-0.99	VCl-H6,F16	0.00	-0.18
VxC-H11,F16	-0.01	-0.24	VCl-C2,H12	0.00	-0.71
VxC-C5,H6	-0.01	-0.99	VCl-H7,H11	0.00	-0.52
VxC-H10,H14	-0.01	-0.99	VCl-H6,H10	0.00	-0.88
VCl-C3,H6	-0.01	-0.91	VxC-C5,H6	0.00	-0.82
VxC-C1,H6	-0.01	-0.95	VxC-C3,H6	0.00	-0.31
VCl-H12,H14	-0.01	-0.99	VxC-C2,F16	0.00	-0.77
VCl-H8,H14	-0.01	-0.97	VxC-C1,H11	0.00	-0.97
VxC-H12,F16	-0.01	-0.70	VxC-H11,H14	0.00	-0.88
VCl-H11,O15	-0.01	-0.30	VCl-C2,H6	0.00	-0.86
VCl-H10,H13	-0.01	-0.78	VCl-H10,H12	0.00	-0.94
VCl-H10,O15	-0.01	-0.03	VxC-H8,H11	0.00	-0.99
VxC-H6,H14	-0.01	-0.95	VCl-C3,H7	0.00	-0.98
VxC-H6,H10	-0.01	-0.98	VxC-C4,H8	0.00	-0.99
VxC-H13,H14	-0.01	-0.95	VCl-H7,H8	0.00	-0.93
VCl-C3,H11	-0.01	-0.97	VCl-H8,H14	0.00	-0.98
VCl-C3,H10	-0.01	-0.78	VxC-H7,H14	0.00	-0.99
VCl-H13,H14	-0.01	-0.99	VxC-H8,H12	0.00	-1.00
VCl-H6,H14	-0.01	-0.99	VCl-H9,H13	0.00	-0.33
VxC-C5,H7	-0.01	-0.95	VxC-H7,H8	0.00	-0.53
VxC-H9,F16	-0.01	-0.89	VxC-H6,H12	0.00	-1.00
VCl-C4,H14	-0.01	-0.98	Eintra-H6	0.00	-0.66
VCl-H11,H14	-0.01	-0.99	VCl-H6,H7	0.00	-1.00
VCl-H10,H14	-0.01	-0.99	VCl-H7,H12	0.00	-0.98
VxC-H10,H13	-0.01	-0.99	VxC-C1,H7	0.00	-0.45
VCl-H11,H13	-0.01	-0.92	VxC-H7,H12	0.00	-1.00
VCl-H9,H11	-0.01	-0.96	VCl-H7,H13	0.00	-0.95
VxC-C3,H11	-0.01	-0.92	VCl-C4,H10	0.00	-0.94
VxC-C2,C5	-0.01	-0.65	VxC-C4,H6	0.00	-0.83
VCl-C2,H14	-0.02	-1.00	VCl-C4,H7	0.00	-0.97
VxC-H6,H8	-0.02	-0.90	VCl-H7,H10	-0.01	-0.99
VxC-C2,H10	-0.02	-0.85	VxC-C2,H12	-0.01	-1.00
VxC-C5,H14	-0.02	-0.98	VCl-H12,F16	-0.01	-0.95
VCl-C4,H9	-0.02	-0.93	VxC-C2,H6	-0.01	-0.55
VCl-C5,H7	-0.02	-0.42	VxC-C5,H7	-0.01	-0.99
VxC-H9,H10	-0.02	-0.92	VxC-H6,O15	-0.01	-0.92
VxC-C5,H10	-0.02	-0.70	VxC-C3,H11	-0.01	-0.76
VCl-H6,F16	-0.02	-0.98	VxC-H10,H13	-0.01	-0.95

VCl-H6,O15	-0.02	-0.83	VxC-C3,H7	-0.01	-0.63
VxC-C5,H12	-0.03	-0.98	VCl-C1,C4	-0.01	-0.59
VCl-C4,H11	-0.03	-0.98	VxC-H7,F16	-0.01	-0.95
VCl-H9,H13	-0.03	-0.98	VxC-H9,F16	-0.01	-0.98
VxC-H11,H13	-0.03	-0.99	VxC-H11,H12	-0.01	-1.00
VCl-H10,F16	-0.04	-0.37	VxC-H6,H8	-0.01	-0.99
VxC-C2,H11	-0.04	-0.87	VxC-H9,H10	-0.01	-0.98
VxC-C3,C5	-0.04	-0.97	VCl-H13,H14	-0.01	-0.99
VCl-C3,H13	-0.04	-0.99	Eintra-C4	-0.01	-0.43
VxC-H9,O15	-0.04	-0.64	VxC-C2,H9	-0.01	-0.78
VCl-H8,F16	-0.04	-0.91	VCl-H9,H14	-0.01	-0.91
VxC-H12,O15	-0.04	-0.99	VCl-C1,H12	-0.02	-0.97
VCl-C3,H9	-0.04	-0.98	VCl-C2,F16	-0.02	-0.95
VCl-H12,F16	-0.04	-0.99	VxC-C1,H14	-0.02	-0.61
VxC-C4,H13	-0.04	-0.96	VxC-C2,H10	-0.02	-0.96
VxC-H10,O15	-0.04	-0.81	VxC-C5,H12	-0.02	-0.95
VCl-H9,H10	-0.05	-0.98	Eintra-H14	-0.02	-0.98
VxC-C3,F16	-0.05	-0.99	VxC-C2,C5	-0.03	-0.98
VxC-C5,H11	-0.05	-0.98	VCl-H6,O15	-0.03	-0.92
Eintra-H7	-0.05	-0.68	VxC-H7,H10	-0.03	-0.90
VCl-H11,F16	-0.05	-0.99	VxC-H14,O15	-0.03	-0.79
VxC-H13,O15	-0.06	-0.52	VxC-H9,O15	-0.04	-0.96
VxC-C3,H9	-0.07	-0.91	VxC-C3,H14	-0.04	-0.83
Eintra-H8	-0.07	-0.95	VCl-C2,H7	-0.04	-0.94
VCl-C5,H10	-0.07	-0.34	VCl-C5,H12	-0.04	-0.99
VxC-C3,H10	-0.08	-0.98	VCl-C4,C5	-0.04	-0.63
VxC-H7,H9	-0.08	-0.98	VCl-H8,F16	-0.04	-0.98
VxC-C2,C4	-0.09	-0.83	VxC-C2,H7	-0.05	-0.83
VxC-H7,H11	-0.10	-0.90	VxC-O15,F16	-0.05	-0.30
VxC-H8,O15	-0.10	-0.98	VxC-C1,H8	-0.05	-1.00
VCl-C2,F16	-0.10	-0.99	VxC-C2,H11	-0.05	-1.00
Eintra-H9	-0.11	-0.70	VxC-H8,H9	-0.05	-0.96
VCl-C3,H14	-0.11	-0.99	VxC-H13,F16	-0.05	-1.00
VxC-C2,H9	-0.11	-0.99	VxC-H12,H13	-0.05	-1.00
VxC-C4,H7	-0.11	-0.90	VCl-H11,F16	-0.06	-1.00
VxC-H9,H14	-0.11	-0.97	VCl-H14,F16	-0.06	-0.96
VCl-H12,O15	-0.12	-1.00	VCl-H8,O15	-0.07	-0.98
VCl-C1,C3	-0.12	-0.99	VxC-C2,C3	-0.07	-0.97
VxC-C4,F16	-0.13	-0.99	VCl-C3,F16	-0.07	-0.99
VxC-C5,F16	-0.13	-0.74	VxC-H7,H11	-0.08	-1.00
Eintra-H10	-0.14	-0.68	VxC-C4,H7	-0.08	-1.00
VxC-C1,H14	-0.15	-0.99	VCl-C1,H7	-0.09	-0.97
VxC-C1,H8	-0.16	-0.99	VCl-C2,O15	-0.10	-0.98
VCl-C1,H14	-0.16	-1.00	VxC-H13,O15	-0.10	-0.93
VxC-H13,F16	-0.17	-0.99	VxC-C4,H12	-0.10	-0.98
VCl-C1,H13	-0.17	-0.99	VCl-C3,O15	-0.11	-0.99
VCl-C4,O15	-0.18	-0.94	VCl-C5,H10	-0.11	-0.93
VCl-H8,O15	-0.18	-0.96	VCl-C4,O15	-0.12	-0.87

VCl-C4,F16	-0.22	-0.99	VxC-C3,O15	-0.14	-0.89
VCl-C5,O15	-0.22	-0.22	VxC-C2,C4	-0.14	-1.00
VxC-C3,C4	-0.23	-1.00	VCl-C5,H7	-0.15	-0.99
VxC-C1,C3	-0.25	-0.99	VCl-H9,F16	-0.16	-0.94
VxC-C2,C3	-0.29	-0.94	VCl-H11,O15	-0.16	-0.99
Eintra-H14	-0.30	-0.98	VCl-H13,F16	-0.18	-0.97
VCl-C5,H14	-0.31	-1.00	VxC-C1,C2	-0.19	-0.93
VCl-C3,C5	-0.34	-0.99	VCl-C1,H10	-0.21	-0.88
Eintra-C3	-0.45	-0.99	Eintra-C1	-0.22	-0.46
VxC-O15,F16	-0.47	-0.94	VxC-C1,H9	-0.25	-0.96
VxC-C4,C5	-0.48	-0.99	VxC-H11,F16	-0.26	-1.00
VCl-C2,O15	-0.50	-1.00	VxC-C3,C4	-0.26	-0.98
VCl-C5,H9	-0.62	-0.98	VxC-C5,H13	-0.28	-1.00
VCl-C5,F16	-0.71	-0.55	VCl-H9,O15	-0.32	-0.88
VCl-C1,H9	-0.75	-0.99	VxC-C5,O15	-0.47	-0.88
VCl-C5,H13	-0.78	-0.96	VCl-H13,O15	-0.51	-0.97
VCl-C1,O15	-0.91	-0.65	VCl-C1,C5	-0.59	-0.99
VCl-C1,C5	-1.03	-0.88	Eintra-C5	-1.27	-0.97
Eintra-O15	-1.11	-0.99	Eintra-F16	-1.39	-0.95
VxC-C1,C2	-1.76	-1.00	VCl-C5,O15	-3.34	-0.93
